# Extracellular Vesicles Derived from Human Umbilical Cord Mesenchymal Stem Cells Protect Liver Ischemia/Reperfusion Injury by Reducing CD154 Expression on CD4+ T Cells via CCT2

**DOI:** 10.1002/advs.201903746

**Published:** 2020-08-20

**Authors:** Jun Zheng, Tongyu Lu, Chaorong Zhou, Jianye Cai, Xiaomei Zhang, Jinliang Liang, Xin Sui, Xiaoyan Chen, Liang Chen, Yao Sun, Jiebin Zhang, Wenjie Chen, Yingcai Zhang, Jia Yao, Guihua Chen, Yang Yang

**Affiliations:** ^1^ Department of Hepatic Surgery and Liver Transplantation Center, Guangdong Key Laboratory of Liver Disease Research Guangdong Province Engineering Laboratory for Transplantation Medicine The Third Affiliated Hospital of Sun Yat‐sen University 600 Tianhe Road Guangzhou 510630 China; ^2^ Department of Hepatic Surgery and Liver Transplantation Center The Third Affiliated Hospital of Sun Yat‐sen University 600 Tianhe Road Guangzhou 510630 China; ^3^ The Second Affiliated Hospital of Guangzhou Medical University Guangzhou 510630 China; ^4^ Organ Transplantation Research Center of Guangdong Province Key Laboratory of Liver Disease Biotherapy and Translational Medicine of Guangdong Higher Education Institutes The Third Affiliated Hospital of Sun Yat‐sen University 600 Tianhe Road Guangzhou 510630 China; ^5^ Surgical ICU The Third Affiliated Hospital of Sun Yat‐sen University 600 Tianhe Road Guangzhou 510630 China; ^6^ Biological Treatment Center The Third Affiliated Hospital of Sun Yat‐sen University 600 Tianhe Road Guangzhou 510630 China

**Keywords:** CCT2, CD4+ T cells, extracellular vesicles, liver ischemia/reperfusion injury, mesenchymal stem cells

## Abstract

As a cause of postoperative complications and early hepatic failure after liver transplantation, liver ischemia/reperfusion injury (IRI) still has no effective treatment during clinical administration. Although the therapeutic potential of mesenchymal stem cells (MSCs) for liver IRI has been previously shown, the underlying mechanisms are not completely clear. It is accepted that MSC‐derived extracellular vesicles (MSC‐EVs) are newly uncovered messengers for intercellular communication. Herein, it is reported that umbilical cord‐derived MSCs (UC‐MSCs) improve liver IRI in mice through their secreted EVs. It is also visualized that UC‐MSC‐EVs mainly concentrate in liver after 6 h of reperfusion. Furthermore, UC‐MSC‐EVs are found to significantly modulate the membranous expression of CD154 of intrahepatic CD4+ T cells, which is an initiation of inflammatory response in liver and can aggravate liver IRI. Mechanistically, protein mass spectrum analysis is performed and it is revealed that Chaperonin containing TCP1 subunit 2 (CCT2) enriches in UC‐MSC‐EVs, which regulates the calcium channels to affect Ca^2+^ influx and suppress CD154 synthesis in CD4+ T cells. In conclusion, these results highlight the therapeutic potential of UC‐MSC‐EVs in attenuating liver IRI. This finding suggests that CCT2 from UC‐MSC‐EVs can modulate CD154 expression of intrahepatic CD4+ T cells during liver IRI through the Ca^2+^‐calcineurin‐NFAT1 signaling pathway.

## Graphical abstract

Mesenchymal stem cell‐derived extracellular vesicles (MSC‐EVs) attenuate liver ischemia/reperfusion injury via Chaperonin Containing TCP1 subunit 2 (CCT2) regulating the activity of calcium channels, Orai1, of CD4+ T cells and subsequent Ca^2+^‐calcineurin‐nuclear factor of the activated T cells 1 (NFAT1) pathway, which inhibits CD154 synthesis and expression on CD4+ T cells.

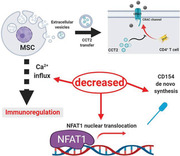

## 1 Introduction

Orthotopic liver transplantation (OLT) is still the most effective treatment for the patients with end‐stage of liver diseases and hepatic malignant tumor. However, liver ischemia/reperfusion injury (IRI), characterized by inflammatory cascade and hepatocellular apoptosis, is an inevitable problem, which is a risk factor predisposing recipients to an early hepatic failure and causing high rate of morbidity and mortality.^[^ ^1^, ^2^, ^3^ ^]^ As the severe shortage of donor organs become the most challenging problem in transplantation, the issue of how to attenuate liver IRI is paid an increasing concern worldwide.^[^ ^4^ ^]^ Despite there were several advances in alleviating this pathological injury, many of them remain not suitable for clinically translational application.^[^ ^5^ ^]^

With the capabilities to modulate immune reactivity and promote regeneration, mesenchymal stem cells (MSCs) have been identified as a promising therapy for inflammation‐related diseases in animal studies and in clinical researches.^[^ ^6^, ^7^ ^]^ In the previous study, we have also revealed the beneficial effect of MSCs on preventing liver IRI.^[^ ^8^ ^]^ Recently, mounting evidence suggested that the biological function of MSCs mainly depends on their secreted extracellular vesicles (EVs) that play important roles of cell micro‐communication and bioinformation transportation by inner proteins, microRNA and lncRNA.^[^ ^9^, ^10^, ^11^ ^]^ With the advantages of avoiding the risks of tumorigenicity, pulmonary embolism and allo‐immune response as well as facilitating transplantation and storage, MSC‐EVs exhibit potential clinical application value, which might replace MSCs.^[^ ^12^ ^]^ Recent studies have showed that MSC‐EVs could mimic the capability of MSCs to modulate the activity of a wide range of immune cells, including T cells, B cells, macrophages, and NK cells.^[^ ^13^ ^]^ Our previous study also demonstrated that similar to MSCs, MSC‐EVs could improve liver IRI by suppressing neutrophil inflammatory response and delivering MnSOD into hepatocytes to alleviate oxidative stress, suggesting that MSC‐EVs serve as a novel potential therapeutic approach for liver IRI.^[^ ^14^ ^]^ However, the underlying mechanisms for these phenomena still need to be fully elucidated.

Previous studies have demonstrated a critical role of CD4+ T cells in initiating liver inflammatory response against IRI. After reperfusion, antigen‐specific CD4+ T cells obviously proliferate, infiltrate, and accumulate in the post‐ischemic tissue.^[^ ^15^ ^]^ The activated CD4+ T cells high express CD154 (CD40‐ligand), which further stimulates immune response and increases platelet via CD40/CD154 interaction to aggravate hepatocellular injury.^[^ ^16^ ^]^ CD154 is a 32–39 kDa cell surface protein, which is a member of the tumor necrosis factor (TNF) superfamily.^[^ ^17^, ^18^ ^]^ In normal condition, the baseline expression of CD154 is low. Since CD4+ T cells activation, a large amount of CD154 synthesize constantly and express on the cell surface, which binds CD40 on B cells, NK cells, dendric cells, macrophages, basophils, and eosinophils and activates downstream transcription factors to induce cytokine production.^[^ ^19^, ^20^, ^21^ ^]^ In addition, CD154 is also a short‐lived protein, which is degraded in a short time. Therefore, the therapeutic approaches for governing CD154 expression on the intrahepatic CD4+ T cells in the early period are necessary for improving IRI and reducing the complications and mortality rate during OLT.

Here, we hypothesized that MSC‐EVs exert hepatoprotective roles via downregulating CD154 expression on the intrahepatic CD4+ T cells. In the further mechanistic investigation, we targeted to Chaperonin containing TCP1 subunit 2 (CCT2) in MSC‐EVs transferring into CD4+ T cells that regulated calcium influx/nuclear factor of activated T cells 1 (NFAT1) signaling pathway and finally affected CD154 synthesis and expression. We used MSC‐EVs and CCT2 knockdown MSC‐EVs in in vivo and in vitro researches to test our hypothesis.

## 2 Results

### 2.1 UC‐MSCs Improve Liver IRI and Suppress CD154 Expression on Intrahepatic CD4+ T Cells

**Figure 1:**
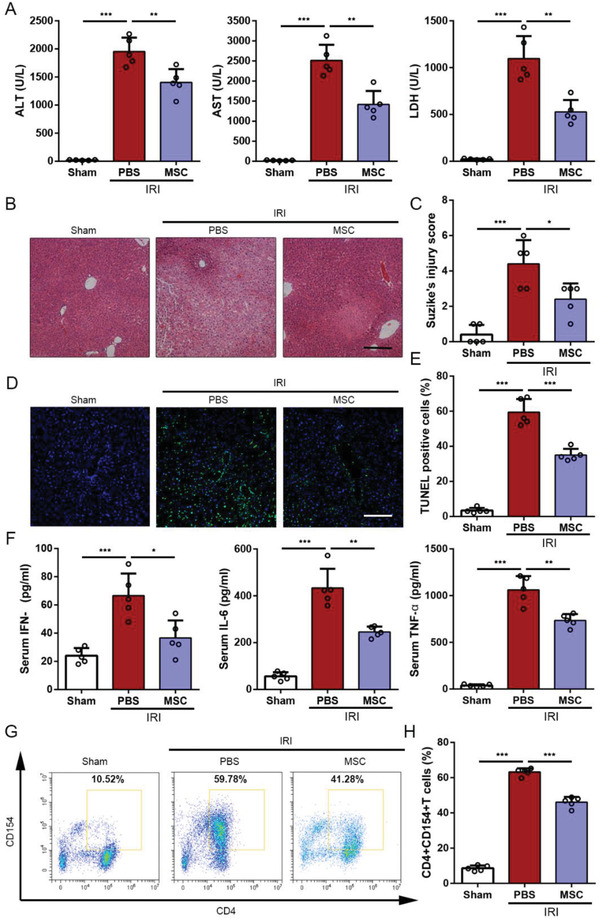
Effects of UC‐MSCs treatment on attenuating injury and suppressing CD154 expression on intrahepatic CD4+ T cells in the mouse liver IRI model. Mice that underwent liver IRI and were treated with UC‐MSCs or PBS were sacrificed at 6 h after reperfusion. A) Serum ALT, AST, and LDH from normal control (Sham group), liver IRI mice and UC‐MSCs‐treated liver IRI mice were detected at 6 h after reperfusion. The data are expressed as the means ± SEMs (*n* = 5 per group). B) Representative sections of livers stained with H&E from three groups after different treatments (bar = 200 µm). C) The severity of liver injury was evaluated from histological section and scored according to Suzike's injury criteria. The data are expressed as the means ± SEMs (*n* = 5 per group). D,E) Representative sections from each group stained with fluorescent TUNEL (bar = 200 µm). Statistical analyses of the percent of TUNEL positive cells in each field. Data are presented as the means ± SEM (*n* = 5 mice per group). F) The levels of IFN‐*γ*, IL‐6, and TNF‐*α* in serum from each group were measured using ELISA assay. Data are presented as the means ± SEM (*n* = 5 mice per group). G,H) Flow cytometry analyses of CD4+CD154+ T cells in intrahepatic CD3‐positive mononuclear cells of each treatment group. Statistical analyses of the percent of CD4+CD154+ cells in each group. Data are presented as the means ± SEM (*n* = 5 mice per group). *p < 0.05, **p < 0.01, ***p < 0.001 (all *p* values were obtained by one‐way ANOVA).

The severity of liver IRI was determined according to serum hepatic enzymes, including alanine aminotransferase (ALT), aspertate aminotransferase (AST), and lactate dehydrogenase (LDH), and liver histopathological changes. As shown in **Figure** **1**A, ALT, AST, and LDH significantly increased in 6 h of reperfusion compared to the sham group, and elevated levels of serum hepatic enzymes were markedly reversed after umbilical cords‐derived MSCs (UC‐MSCs) treatment. Histopathology demonstrated obvious hepatocellular injury from the IRI mice, as evidenced by disorder of hepatic lobules, tissue necrosis, and increased infiltration of inflammatory cells (Figure 1B). And the severity of Suzike's score was significantly reduced after treated with UC‐MSCs (Figure 1C). Furthermore, the terminal deoxynucleotidyl transferase dUTP nick end labeling (TUNEL) assay was recognized as an indicator of cell apoptosis, and the results showed that TUNEL‐positive cells were significantly increased in the model mice, transplanted UC‐MSCs markedly decreased hepatocellular apoptosis after 6 h reperfusion (Figure 1D,E).

To detect the differential immune status in each group, we measured the protein levels of IFN‐*γ*, IL‐6, and TNF‐*α* in serum by enzyme‐linked immunosorbent assay ELISA. As shown in Figure 1F, compared to the sham group, IFN‐*γ*, IL‐6, and TNF‐*α* were significantly increased in serum in liver IRI mice, whereas UC‐MSC obviously suppressed the levels of these cytokines. Growing evidence demonstrated that the increased levels of TNF‐*α* and IFN‐*γ* in the early periods of IRI were mainly secreted by CD4+ T cells. Moreover, we isolated intrahepatic mononuclear cells, analyzed by flow cytometry and found that elevating expression of CD154 on intrahepatic CD4+ T cells, which induced by liver IRI, was gradually reversed after the administration of UC‐MSCs (Figure 1G,H and Figure S1A,B, Supporting Information). Additionally, we also evaluated the levels of macrophages (CD68‐positive cells) infiltrating in liver tissue from each group. As shown in Figure S1C in the Supporting Information, CD68‐positive cells in the UC‐MSC group were significantly lower than that in the phosphate buffer saline (PBS) group. These findings confirmed the potential therapeutic effect of UC‐MSCs on liver IRI, which is similar to our previous study,^[^ ^8^, ^22^ ^]^ and further showed that UC‐MSCs played an important role in modulating intrahepatic CD4+ T cell functions, including cytokines secretion and CD154 expression.

### 2.2 CD4+ T Cells are Pivotal for the Initiation of Inflammatory Response in Liver IRI

To verify the role of CD4+ T cells in pro‐inflammatory response against liver IRI, we additionally used CD4 depleting Ab (GK1.5) to scavenge CD4+ T cells in vivo.^[^ ^15^ ^]^ These mice pre‐treated with GK1.5 were subjected to liver IRI, and hepatic damage were detected at 6 h of reperfusion. Depleting CD4+T cells significantly protected from IRI, as evidenced by their serum hepatic enzymes, including ALT, AST, and LDH, were lower, liver pathological changes were more effectively improved and architecture was better protected, compared with that in wide‐type (WT) mice (Figure S2A,B, Supporting Information). Furthermore, we also investigated the inflammatory status in these two groups and showed that the infiltration of macrophages in liver tissue and the levels of pro‐inflammatory cytokines, IFN‐*γ*, IL‐6 and TNF‐*α*, in serum were also lower in the GK1.5 group than that in the WT group (Figure S2C,D, Supporting Information).

### 2.3 UC‐MSC‐CM Suppresses CD4+ T Cells Inflammatory Cytokines Production and CD154 Expression In Vitro

Next, we aimed to clarify whether the EVs exhibited immunoregulative potential described for UC‐MSCs paracrine secretion. First, CD4+ T cells for in vitro experiments were isolated from spleen in mice and purified using a MicroBeads UltraPure kit. The results from flow cytometry analysis confirmed the purity of CD4+ T cells was more than 95% (Figure S3A, Supporting Information). Then, we, respectively, added two distinct medium, conditioned medium (CM) and conditioned medium that removes EVs fraction through ultracentrifugation (ECM), to CD4+ T cells to compare their ability on modulating T cell response. As shown in Figure S4BC in the Supporting Information, UC‐MSC‐CM significantly reduced CD154 expression on CD4+ T cells stimulated by phorbol myristate acetate (PMA) and ionomycin, while treatment with ECM (conditioned medium that removes EVs fraction through ultracentrifugation) reversed this effect. In addition, we also quantified the secretion function of CD4+ T cells which is modulated by UC‐MSC‐CM. Both TNF‐*α* and IFN‐*γ* in CD4+ T cells from each group were detected by quantitative reverse transcription polymerase chain reaction (RT‐qPCR), and the results showed that compared with the control group, PMA and ionomycin treatment increased the concentrations of these cytokines and the addition of UC‐MSC‐CM significantly decreased the expression of these cytokines in CD4+ T cells (Figure S4D, Supporting Information). Notably, removing EVs fraction reversed the effect of UC‐MSC‐CM on regulating the secretory function of CD4+ T cells. These results suggested that EVs derived from UC‐MSCs may be involved in CD4+ T cells immunomodulation after PMA and ionomycin stimulation.

### 2.4 UC‐MSC‐EVs Improve Liver IRI and Regulate CD154 Expression on CD4+ T Cells In Vitro and In Vivo

**Figure 2:**
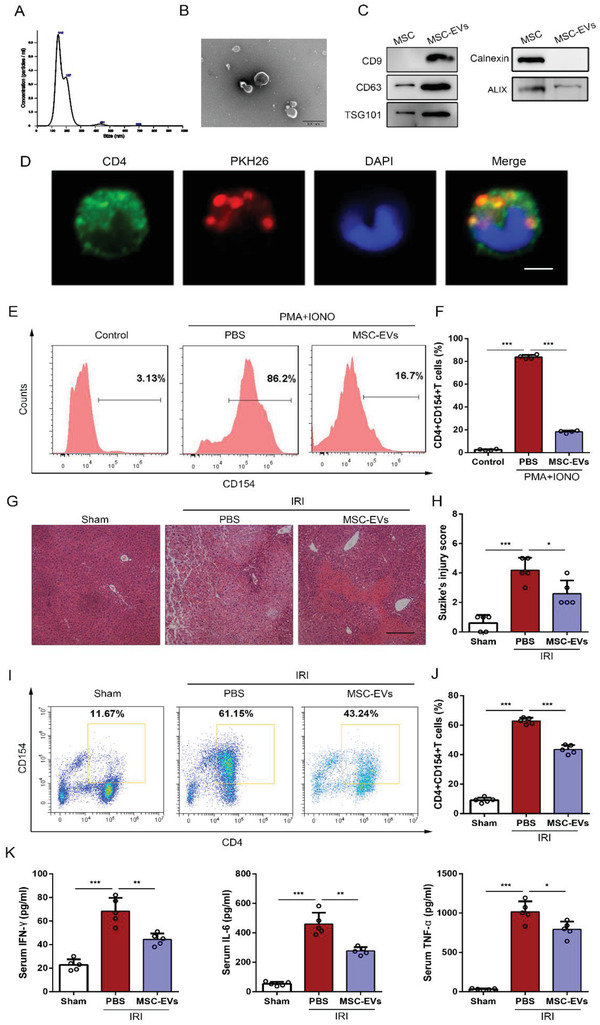
UC‐MSC‐EVs improve liver IRI and regulate CD154 expression on CD4+ T cells in vitro and in vivo. Characterization of harvested UC‐MSC‐EVs. A) Nanoparticle tracking analysis was performed to detect the diameter quantitation of UC‐MSC‐EVs. B) The spheroid morphology and size of UC‐MSC‐EVs were investigated under SEM. Original scale bar: 0.2 µm. C) Western blot analysis was conducted to detect the specific vesicle‐related markers CD63, CD9, ALIX, TSG101, and calnexin in UC‐MSC‐EVs. D) The CD4+ T cells were stimulated by PMA and ionomycin and treated with PKH26‐labeled UC‐MSC‐EVs for 6 h, a representative fluorescence image of UC‐MSC‐EVs (red) uptake in CD4+ T cells (green) was detected and photographed using confocal microscopy (bar = 2 µm). In vitro experiments, primary CD4+ T cells isolated from the spleen of mice were stimulated by PMA and ionomycin, and co‐cultured with PBS or UC‐MSC‐EVs. E) Flow cytometry analyses of CD154 expression on CD4+ T cells of each treatment group. F) Quantification of membranous CD154 expression of CD4+ T cells. The data are presented as the means ± SEM (*n* = 3 per group). Mice that underwent liver IRI and were treated with UC‐MSC‐EVs or PBS were sacrificed at 6 h after reperfusion. G) Representative sections of livers stained with H&E from three groups after different treatments (bar = 200 µm). H) The severity of liver injury was evaluated from histological section and scored according to Suzike's injury criteria. The data are expressed as the means ± SEMs (*n* = 5 per group). I,J) Flow cytometry analyses of CD4+CD154+ T cells in intrahepatic CD3‐positive mononuclear cells of each treatment group. Statistical analyses of the percent of CD4+CD154+ T cells in each group. Data are presented as the means ± SEM (*n* = 5 mice per group). K) The levels of IFN‐*γ*, IL‐6, and TNF‐*α* in serum from each group were measured using ELISA assay. Data are presented as the means ± SEM (*n* = 5 mice per group). *p < 0.05, **p < 0.01, ***p < 0.001 (all *p* values were obtained by one‐way ANOVA).

EVs were harvested from UC‐MSC‐CM by ultracentrifugation and characterized using a nanoparticle tracking analysis (NTA), Western blot analysis, and transmission electron microscopy (TEM). Consistent to our previous study, the results confirmed that the size of these vesicles ranged from 30 to 150 nm (**Figure** **2**A). By TEM, we observed round‐shaped particles compassed by a bilayer membrane (Figure 2B).^[^ ^14^ ^]^ Western blot analysis showed that EV‐related markers, including CD63, CD9 TSG101, and ALIX, were positively expressed and calnexin was negatively expressed in EVs (Figure 2C).

To detect if UC‐MSC‐EVs could be taken by CD4+ T cells, PKH26‐labeled UC‐MSC‐EVs were incubated with CD4+ T cells for 6 h. The results from confocal images showed that UC‐MSC‐EVs (red) were situated in the cytoplasm of CD4+ T cells (green), suggesting that CD4+ T cells could take in UC‐MSC‐EVs (Figure 2D). To investigate the effects of UC‐MSC‐EVs on regulating CD154 expression on CD4+T cells, we also treated primary CD4+ T cells with UC‐MSC‐EVs, which were stimulated by PMA and ionomycin simultaneously, for 6 h to detect the transient role of EVs. Flow cytometry analysis revealed that treatment with UC‐MSC‐EVs significantly suppressed CD154 synthesis and membranous expression of CD4+ T cells, compared with the cells treated with PMA and ionomycin alone (Figure 2E,F). In addition, we also showed from the results of RT‐qPCR that UC‐MSC‐EVs remarkably inhibited TNF‐*α* and IFN‐*γ* production of CD4+ T cells (Figure S5A, Supporting Information).

We further assessed the hepatoprotective effects of UC‐MSC‐EVs in IRI mice. We found that compared with the PBS group, the mice treated with UC‐MSC‐EVs had obviously lower levels of ALT, AST, and LDH at 6 h of reperfusion (Figure S5B, Supporting Information). Hematoxylin and eosin (H&E) staining of liver tissue in each group showed that similar to the effect of UC‐MSC, the administration of UC‐MSC‐EVs significantly inhibited hepatocellular necrosis and reduced Suzike's score (Figure 2G,H). TUNEL staining revealed that the number of apoptotic cells was significantly reduced in the UC‐MSC‐EVs treatment group (Figure S5C, Supporting Information). Furthermore, we detected the functions of intrahepatic CD4+ T cells from different treatments and found that the expression of CD154 on intrahepatic CD4+ T cells was significantly decreased as well as the levels of IFN‐*γ*, IL‐6, and TNF‐*α* in serum were obviously reduced after administrated with UC‐MSC‐EVs, compared with the group treated with PBS (Figure 4I–K and Figure S5D,E, Supporting Information). As the role of CD4+ T cells in the initiating inflammatory response during liver IRI, we subsequently counted the number of macrophages in liver tissues from each group. As shown in Figure S5F in the Supporting Information, CD68‐positive cells in the UC‐MSC‐EV group were significantly lower than that in the PBS group, which may partially attribute to regulate CD4+ T cells functions by UC‐MSC‐EVs.

### 2.5 The Biodistribution of UC‐MSC‐EVs in Liver IRI Model

**Figure 3:**
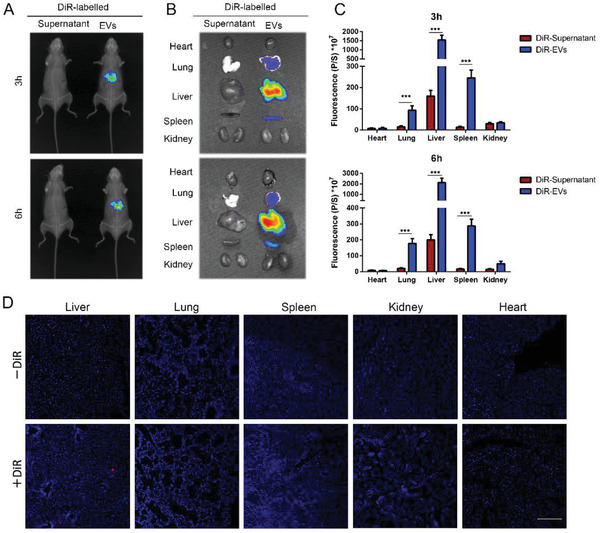
The biodistribution of UC‐MSC‐EVs in a mouse liver IRI model. Biodistribution of UC‐MSC‐EVs labeled by DiR was intravenously transfused through caudal vein after the mice IRI model conducted. A) Representative IVIS images of 3 and 6 h post‐injection of mice liver IRI model injected i.v. with DiR‐labeled UC‐MSC‐EVs (right) or DiR supernatant‐treated control (left) in vivo. B) Representative IVIS images of five different organs collected at 3 and 6 h following i.v. transfusion of DiR‐labeled UC‐MSC‐EVs or DiR supernatant, respectively. C) Quantification of the fluorescent intensity in five different organs including the heart, lung, liver, spleen, and kidney at 3 and 6 h following administration of DiR‐labeled UC‐MSC‐EVs or DiR supernatant. The data are expressed as the means ± SEMs (*n* = 5 per group). D) Representative fluorescent sections of heart, lung, liver, spleen, and kidney tissue to detect the DiR‐labeled UC‐MSC‐EVs biodistribution (red) were photographed using confocal microscopy (bar = 400 µm). *p < 0.05, **p < 0.01, ***p < 0.001 (all *p* values were obtained by one‐way ANOVA).

Moreover, we detected the biodistribution of transfused UC‐MSC‐EVs in liver IRI model. In this setting, DiR (1,1‐dioctadecyl‐3,3,3,3‐tetramethy‐lindotricarbocyanine iodide) was utilized to labeled UC‐MSC‐EVs, and 100 µg/100 µL DiR‐labeled UC‐MSC‐EVs were administrated via peripheral vein after reperfusion. The mice from each experimental group were taken in vivo fluorescent imaging after 3 and 6 h after the EVs injection, and the results showed that DiR fluorescence was mainly localized in the liver of IRI mice (**Figure** **3**A). Then, the mice were sacrificed, and freshly dissected tissues, including heart, lung, liver, spleen, and kidneys, were harvested and imaged in vitro for the subsequent experiments. Consistent with previous studies, EVs were mostly detected in the liver and spleen, and little in lung (Figure 3B,C). These findings also affirmed in tissue sections that DiR‐labeled UC‐MSC‐EVs were enriched in the livers of IRI mice (Figure 3D).

### 2.6 UC‐MSC‐EVs Regulate the Expression of CD154 via Ca^2+^‐Calcineurin‐NFAT1 Signaling Pathway in CD4+ T Cells

**Figure 4:**
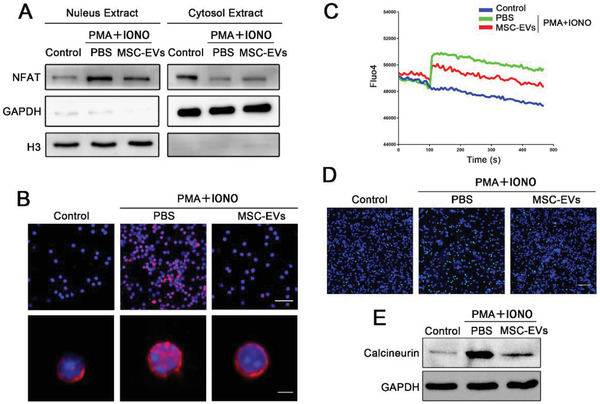
UC‐MSC‐EVs regulate the expression of CD154 via Ca^2+^‐calcineurin‐NFAT1 signaling pathway in CD4+ T cells. Primary CD4+ T cells isolated from the spleen of mice were stimulated by PMA and ionomycin, and co‐cultured with PBS or UC‐MSC‐EVs. A) The levels of NFAT1 in the cytosolic and nuclear fractions were, respectively, detected by Western blotting assay. B) The location of NFAT1 (fluorescent red) in CD4+ T cells was visualized using confocal microscopy. The nucleus was stained with DAPI (fluorescent blue) (upper bar = 30 µm; lower bar = 2 µm). C) Representative curve of Ca^2+^ influx dynamics in PBS‐ and UC‐MSC‐EVs‐treated CD4+ T cells using Fluo4 fluorescence. D) The fluorescence of Fluo4 (fluorescent green) was investigated to measure the level of Ca^2+^ influx in CD4+ T cells in each group (bar = 60 µm). E) The expression of calcineurin in CD4+ T cells in each group was determined by Western blot analysis. The average intensity of the band in the Western blots was quantified using GAPDH as an internal reference. *p < 0.05, **p < 0.01, ***p < 0.001 (all *p* values were obtained by one‐way ANOVA).

Furthermore, we explored the potential mechanism how UC‐MSC‐EVs regulate the synthesis and expression of CD154 on CD4+ T cells. Previous study showed that activation‐induced CD154 expression on CD4+ T cells was depended on signal transducer and activator of transcription 5 (STAT5) to bind the CD154 transcriptional promoter.^[^ ^23^ ^]^ In addition, Crist et al. measured both human and mouse samples and demonstrated that NFAT modulates CD154 expression in megakaryocytes.^[^ ^24^ ^]^ However, Wu et al. revealed that hydroxychloroquine inhibits CD154 expression in CD4+ T cells through regulating NFAT1 signaling.^[^ ^25^ ^]^ Therefore, we speculated in this study that the effect of UC‐MSC‐EVs on suppressing CD154 expression might be partially due to blocking the activation of NFAT1 or STAT5. The results from Western blot analysis showed that compared with the control group, PMA and ionomycin stimulation led to NFAT1 translocation into the nucleus, which presented that expression of NFAT1 significantly increased in the nuclear fraction and decreased in the cytoplasmic fraction, while UC‐MSC‐EVs remarkably reversed these changes (**Figure** **4**A). However, we also found that the increased p‐STAT5 after PMA and ionomycin stimulation did not obviously change upon UC‐MSC‐EVs treatment (Figure S6A, Supporting Information). Thus, these results revealed that UC‐MSC‐EVs treatment inhibits membranous CD154 expression of CD4+ T cells through NFAT1 signaling pathway, but not STAT5. To affirm that, we subsequently performed immunocytochemistry analysis. Through confocal microscopy, we observed that NFAT1 (red fluorescence) remained in the cytoplasm without any treatment. PMA and ionomycin could induce an increased nuclear translocation of NFAT1, and lesser NFAT1 nucleus translocation was investigated after adding UC‐MSC‐EVs (Figure 4B).

NFAT1 is a calcineurin‐dependent protein and identified as a secondary messenger of the Ca^2+^ signaling pathway, which is highly sensitive to the concentration of cytosolic Ca^2+^.^[^ ^26^, ^27^, ^28^, ^29^ ^]^ Previous studies have demonstrated that modulated free Ca^2+^ influx could effectively affect CD4+ T cells functions, including secretion and CD154 expression.^[^ ^30^, ^31^ ^]^ Therefore, we further conducted in vitro experiments to explore whether UC‐MSC‐EVs played a role in inhibiting Ca^2+^ influx in CD4+ T cells after PMA and ionomycin stimulation. The change of intracellular Ca^2+^ concentration was determined by Fluo4 fluorescent dye. According to the data of absorbance values, we found that PMA and ionomycin significantly increased intracellular Ca^2+^ concentration of CD4+ T cells, while addition of UC‐MSC‐EVs caused an obvious decreased Ca^2+^ influx rate (Figure 4C). The fluorescent results also verified these phenomena (Figure 4D). In addition, we detected the protein expression of calcineurin, which participates in various signal transduction pathways modulated by Ca^2+^ and plays an effect on connecting Ca^2+^ and NFAT1, in CD4+ T cells from each group.^[^ ^32^ ^]^ The results showed that the increased expression of calcineurin by PMA and ionomycin was significantly reversed by UC‐MSC‐EVs treatment (Figure 4E). To further validate Ca^2+^ as an important factor in regulating CD4+ T cells function, we additionally used GSK‐5498A, which is an inhibitor of calcium channel, to limit Ca^2+^ influx and deplete Ca^2+^ store in CD4+ T cells.^[^ ^33^ ^]^ Administration of GSK‐5498A led to a markedly reduced Ca^2+^ concentration induced by PMA and ionomycin (Figure S7A, Supporting Information). Furthermore, we also performed flow cytometry analysis and found that GSK‐5498A significantly decreased synthesis and expression of CD154 in CD4+ T cells (Figure S7B,C, Supporting Information). Taken together, the results mentioned above demonstrated that UC‐MSC‐EVs regulate the expression of CD154 via Ca^2+^‐calcineurin‐NFAT1 signaling pathway in CD4+ T cells.

### 2.7 CCT2 Derived from UC‐MSC‐EVs Reduces CD154 Synthesis of CD4+ T Cells by Modulating Its Downstream Target Ca^2+^‐Calcineurin‐NFAT1 Pathway

**Figure 5:**
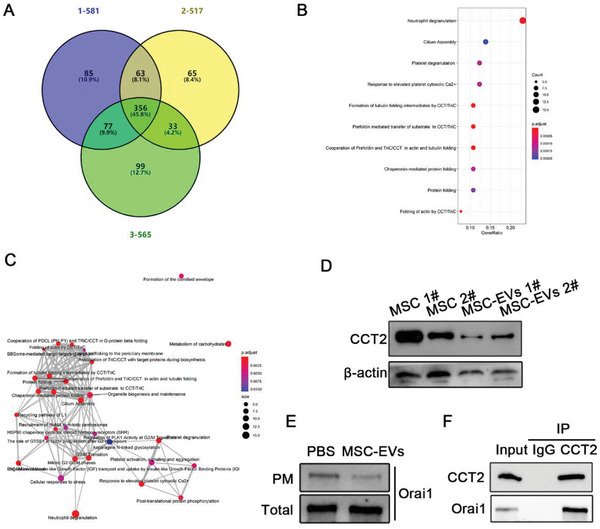
Proteins are identified from UC‐MSC‐EVs. A) A Venn diagram shows a total of proteins from UC‐MSC‐EVs were identified in three independent samples using LC‐MS/MS analysis. B) Dot plot for the analysis of enriched biological pathways. *Y* axis, the enriched biological reaction pathways; *X* axis, GeneRatio. Dot sizes indicate the numbers of proteins found in each enriched pathway. Dot colors indicate the adjusted *p*‐value. C) A network plot for the interaction of enriched biological pathways. Dot sizes indicate the numbers of proteins found in each enriched pathway. Dot colors indicate the adjusted *p*‐value. D) To ascertain the results from the proteomic files, CCT2 expression was evaluated by Western blot analysis. The average intensity of the band in the Western blots was quantified using *β*‐actin as an internal reference. E) The membranous and total expression Orai1 in the CD4+ T cells were, respectively, detected by Western blotting assay. F) Co‐immunoprecipitation with antibody against CCT2 followed by Western blotting using CCT2 and Orai1 antibodies.

EVs are well known to be carried proteins and able to transport protein to target cells, which secreted from host cells, to further modulate the function and viability of recipient cells. To explore the underlying mechanism by which UC‐MSC‐EVs regulate Ca^2+^‐calcineurin‐NFAT1 pathway to decrease CD154 synthesis in CD4+ T cells, we profiled UC‐MSC‐EVs by performing proteomic analysis using high‐resolution liquid chromatography with tandem mass spectrometry (LC‐MS/MS) analysis. Three biological replicates of UC‐MSC‐EVs were included in this analysis. The mean value of proteins which were identified in UC‐MSC‐EVs was 356 (**Figure** **5**A). Then, we used a common online bioinformatic method to analyze the potential proteins that target the calcium channels on CD4+ T cells. Of all the identified proteins, we were specifically intrigued by the enrichment of CCT2 in UC‐MSC‐EVs because of its reported modulation of Ca^2+^ channels trafficking (Figure 5B,C).^[^ ^34^, ^35^ ^]^ Our Western blotting analysis also confirmed that both UC‐MSC‐EVs and UC‐MSCs contained high levels of CCT2 (Figure 5D). In addition, previous study has evidenced that CCT2 could interact and subsequently regulate the subcellular distribution of Ca^2+^ channels (Orai1), which significantly affect Ca^2+^ influx. Thus, to explore the potential mechanism of CCT2 from UC‐MSC‐EVs in regulating Ca^2+^ signaling in CD4+ T cells, we detected the change of total amount and plasma membrane residence of Orai1 after CD4+ T cells uptake of UC‐MSC‐EVs and found that the plasma membrane residence of Orai1 was decreased while the total Orai1 expression was little affected in CD4+ T cells (Figure 5E). Furthermore, the result of CCT2 binding with Orai1 was observed in the co‐immunoprecipitation assay (Figure 5F).

**Figure 6:**
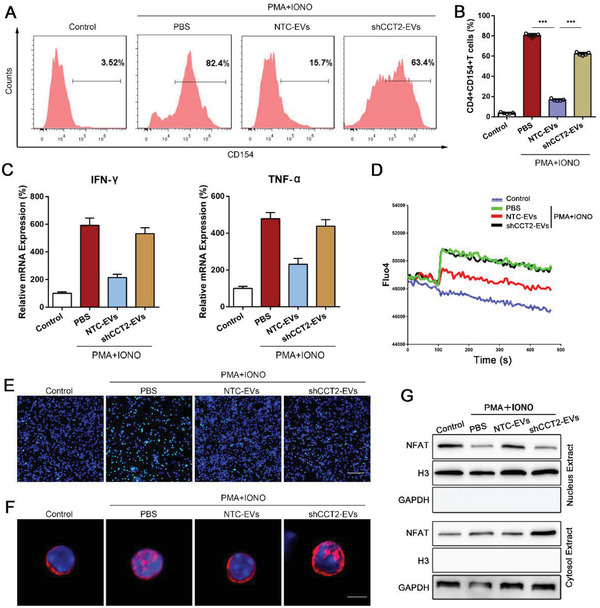
CCT2 derived from UC‐MSC‐EVs reduces CD154 synthesis of CD4+ T cells by modulating its downstream target Ca^2+^‐calcineurin‐NFAT1 pathway. Primary CD4+ T cells isolated from the spleen of mice were stimulated by PMA and ionomycin, and co‐cultured with PBS, MSC‐EVs, or shCCT2‐EVs. A) Flow cytometry analyses of CD154 expression on CD4+ T cells of each treatment group. B) Quantification of membranous CD154 expression of CD4+ T cells. The data are presented as the means ± SEM (*n* = 3 per group). C) The levels of mRNA expression of IFN‐*γ* and TNF‐*α* in CD4+ T cells from each group were determined by RT‐qPCR. The data are presented as mean ± SEM (*n* = 3 per group). D) Representative curves of Ca^2+^ influx dynamics in PBS‐, UC‐MSC‐EVs‐, and shCCT2‐EVs‐treated CD4+ T cells were depicted using Fluo4 fluorescence. E) The fluorescence of Fluo4 (fluorescent green) was investigated to measure the level of Ca^2+^ influx in CD4+ T cells in each group. (Bar = 60 µm). F) The location of NFAT1 (fluorescent red) in CD4+ T cells was visualized using confocal microscopy. The nucleus was stained with DAPI (fluorescent blue) (bar = 2 µm). G) The levels of NFAT1 in the cytosolic and nuclear fractions were detected by Western blot assay. *p < 0.05, **p < 0.01, ***p < 0.001 (all *p* values were obtained by one‐way ANOVA).

To enable further verification of the role of CCT2 in UC‐MSC‐EVs in modulating CD4+ T cells function, lentivirus carrying CCT2‐shRNA was used to knockdown CCT2 in UC‐MSCs. We performed Western blot analysis to confirm that the expression of CCT2 was obviously decreased in UC‐MSCs^shCCT2^, compared with that in the UC‐MSCs (Figure S8A, Supporting Information). And the results from cell counting and multipotential differentiation assay showed that there were no significant differences about the viability and capacities of multi‐differentiation between UC‐MSCs and UC‐MSCs^shCCT2^ (Figure S8B,C, Supporting Information). Subsequently, we harvested UC‐MSC‐EVs^shCCT2^. Consistent with the aforementioned results, UC‐MSC‐EVs reduced membranous CD154 expression of CD4+ T cells, while UC‐MSC‐EVs^shCCT2^ could partially rescue its expression (**Figure** **6**A,B). In addition, the expression of both IFN‐*γ* and TNF‐*α* of CD4+ T cells was higher in the UC‐MSC‐EV^shCCT2^ group than that in the UC‐MSC‐EV group (Figure 6C). We mechanistically showed the results from Fluo4 dye that UC‐MSC‐EVs^shCCT2^ partially reversed the effect of inhibiting Ca^2+^ inflow in CD4+ T cells by UC‐MSC‐EVs (Figure 6D,E). The expression of calcineurin was reduced in UC‐MSC‐EV treated group, and its expression rescued by UC‐MSC‐EVs^shCCT2^ was obviously increased (Figure S9A, Supporting Information). Finally, we also performed both immunofluorescent staining and Western blot analysis and showed that the rate of NFAT1 translocating to the nucleus in CD4+ T cells was significantly upregulated after treated with UC‐MSC‐EVs^shCCT2^, compared with that in the UC‐MSC‐EV group (Figure 6F,G).

### 2.8 CCT2 Derived from UC‐MSC‐EVs Improves Hepatic Damage and Suppresses CD154 Expression on Intrahepatic CD4+ T Cells in Liver IRI Mice

**Figure 7:**
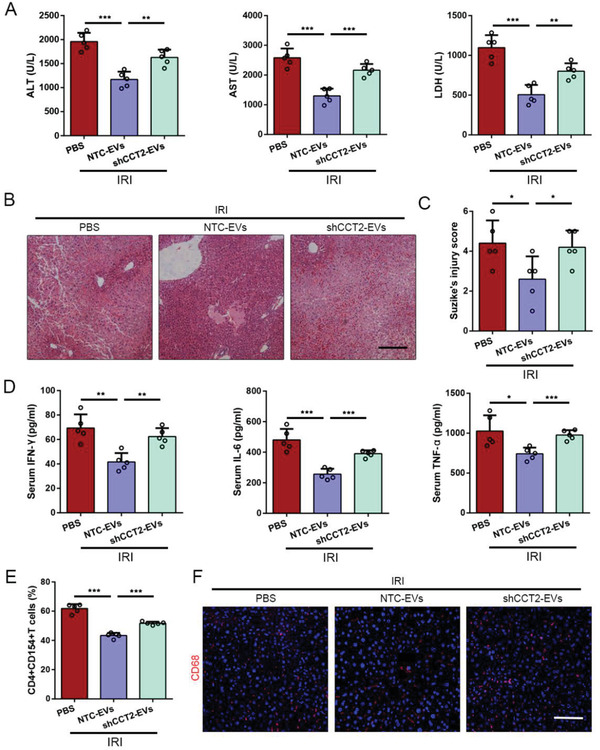
CCT2 derived from UC‐MSC‐EVs improves hepatic damage and suppresses CD154 expression on intrahepatic CD4+ T cells in liver IRI mice. Mice that underwent liver IRI were treated with NTC‐UC‐MSC‐EVs or shCCT2‐UC‐MSC‐EVs and sacrificed at 6 h after reperfusion. A) Serum ALT, AST, and LDH from normal control (Sham group), NTC‐EVs‐treated liver IRI mice, and shCCT2‐EVs‐treated liver IRI mice at 6 h were measured. The data are expressed as the means ± SEMs (*n* = 5 per group). B) Representative sections of livers stained with H&E from three groups after different treatments (bar = 200 µm). C) The severity of liver injury was evaluated from histological section and scored according to Suzike's injury criteria. The data are expressed as the means ± SEMs (*n* = 5 per group). D) The levels of IFN‐*γ*, IL‐6, and TNF‐*α* in serum from each group were measured using ELISA assay. The data are presented as the means ± SEM (*n* = 5 mice per group). E) Statistical analyses of the percent of CD4+CD154+ T cells in intrahepatic mononuclear cells in each group using flow cytometry. The data are presented as the means ± SEM (*n* = 5 mice per group). F) Representative liver sections from each group stained with fluorescent CD68 (red fluorescence) (bar = 200 µm). *p < 0.05, **p < 0.01, ***p < 0.001 (all *p* values were obtained by one‐way ANOVA).

To investigate whether UC‐MSC‐EVs exerted immunoregulative effects in the ischemic mouse liver by CCT2 and its downstream target NFAT1, UC‐MSC‐EVs^shCCT2^ was used in a mouse model of liver IRI. As shown in **Figure** **7**A–C, administration of UC‐MSC‐EVs significantly improved liver injury at 6 h post‐reperfusion, while treatment with UC‐MSC‐EVs^shCCT2^ partially weakened hepatoprotective outcomes, as evidenced by the changes of the levels of serological liver enzymes and hepatic histopathological characterizations (Figure 7A–C). The levels of IFN‐*γ*, IL‐6, and TNF‐*α* were examined in serum in the groups treated with UC‐MSC‐EVs, UC‐MSC‐EVs^shCCT2^, and PBS. The PBS group exhibited high levels of these cytokines in serum of liver IRI mice, and treatment with UC‐MSC‐EVs reduced these pro‐inflammatory factors, whereas administration of UC‐MSC‐EVs^shCCT2^ was shown to reversely increased (Figure 7D). In this section, flow cytometry analysis was also performed to investigate the expression of CD154 on the intrahepatic CD4+ T cells in vivo. As shown in Figure 7E, flow cytometry analysis revealed that treatment with UC‐MSC‐EVs resulted in significantly fewer CD154 expressions on the intrahepatic CD4+ T cells than did treatment with UC‐MSC‐EVs^shCCT2^, which was stimulated by IRI. Finally, we also simultaneously conducted immunofluorescent staining to detect the number of macrophages in liver tissue and found that the reduced count of CD68‐positive cells by UC‐MSC‐EVs treatment was reversely increased after administration of UC‐MSC‐EVs^shCCT2^, which indicated that CCT2 in UC‐MSC‐EVs may limit membranous CD154 expression of intrahepatic CD4+ T cells to affect the activation of subsequent effector cells (Figure 7F).

## 3 Discussion

Stem cell‐based therapies have been widely documented in various preclinical researches and clinical studies, and emerging evidence has also demonstrated that MSCs administration could attenuate liver IRI mainly through their paracrine effects.^[^ ^36^ ^]^ EVs are a pivotal bioactive component of MSCs release and are identified as an alternative to MSCs therapy. In previous study, we had reported that intravenous transfusion of MSC‐EVs is effective in immunomodulation and suppression of oxidative stress in rat liver IRI model.^[^ ^14^ ^]^ In this study, we further provide a novel mechanism on the hepatoprotective roles of MSC‐EVs in modulating CD4+ T cells function that are relevant to the treatment of liver IRI, as well as to the prevention of postoperative complications after LT in general. Results presented here suggest for the first time that MSC‐EVs exert an immunoregulative function in suppressing CD154 synthesis and expression on CD4+ T cells to improve liver IRI. Moreover, this beneficial effect may largely be attributed to CCT2 of EVs, which affected calcium channel, downregulated intracellular calcium concentration, and inhibited transcription factor NFAT1 from translocating to the nucleus.

The function of CD4+ T cells in liver IRI has been fully defined. Through application of recombination activation gene w1 (Rag‐1) knockout mice, Shen et al. found that effector CD4+ T cells reside liver tissue and play an important role in initiating hepatic inflammatory response during IRI.^[^ ^15^ ^]^ In this study, we used CD4+ T cell depleting antibody to also confirm this result. We showed that the severity of hepatic injury and inflammatory response was significantly restored after exhausted CD4+ T cells in vivo by GK1.5. In the exploration of its mechanisms, CD154, expressing on CD4+ T cells, is considered as an indispensable factor for both hepatic injury and liver inflammation against IRI, which were demonstrated by using antibody CD40 or knockout CD154 expression in vivo.^[^ ^37^ ^]^ CD154, a CD40‐ligand, is a tumor necrosis factor (TNF) superfamily and a membrane glycoprotein with a molecular weight of 32–39 kDa.^[^ ^38^ ^]^ Several studies have revealed that CD154 expression obviously increases on the activated CD4+ T cells and interacts with its cognate receptor CD40 to stimulate both adaptive and innate immune systems, including B cells, CD8+ T cells, macrophages, NK cells, and neutrophils.^[^ ^19^ ^]^ In addition, CD154 is a transient protein, which expresses on CD4+ T cells at peak after 6 h stimulation.^[^ ^39^ ^]^ Therefore, therapies aim to inhibit CD154 expression in the early period may be an effective strategy to block inflammatory response and improve liver IRI.

MSCs transfusion has been identified as a promising approach for preventing postoperative complications after LT because they regulate inflammatory response and protect against hepatocellular apoptosis through their paracrine roles. Our previous studies from both preclinical and clinical researches also demonstrated their effects in improving ischemia‐type biliary lesion after LT and attenuating liver IRI.^[^ ^8^, ^40^ ^]^ In the present study, we additionally found that accompanied by the recovery of liver function and liver pathological changes in the IRI model, UC‐MSCs could not only reduce the levels of serological pro‐inflammatory cytokines (IL‐6, TNF‐*α* and IFN‐*γ*), but also suppress membranous CD154 expression of the intrahepatic CD4+ T cells. Mechanistically, recent studies have demonstrated that MSC‐CM was similar to MSCs, which also exhibited roles in immunomodulation and promoting hepatocyte regeneration.^[^ ^11^, ^41^, ^42^ ^]^ And the effects of MSC‐CM on reducing T cells proliferation and inflammatory cytokines production as well as transforming monocytes polarization were attributed to nanosized MSC‐EVs.^[^ ^43^ ^]^ In this setting, we also compared non‐EV CM and fully CM and concluded that MSC‐EVs were proposed as a main bioactive component of MSCs to affect CD154 synthesis of CD4+ T cells. Then, EVs from MSC‐CM were purified by ultracentrifugation for subsequent researches. The expected results may be partially described as a potential mechanism of MSCs to reduce CD154 synthesis and expression on CD4+ T cells, which is attributed to their secreted‐EVs. In in vivo study, we also used MSC‐EVs, which replaced MSCs, to treat a mouse liver IRI model. Consistent to the previous study, MSC‐EVs could attenuate ischemia/reperfusion induced liver injury, modulate inner immune status, and suppress membranous CD154 expression of intrahepatic CD4+ T cells to block the initiation of inflammatory response during liver IRI.

Next, we defined the potential mechanisms by which MSC‐EVs attenuated IRI‐induced membranous CD154 expression of the intrahepatic CD4+ T cells. First, we identified that UC‐MSC‐EVs can rapidly migrate and localize to the injured tissue after systemic transfusion. Previous study has demonstrated that high expression of CD154 on CD4+ T cells of systemic lupus erythematosus (SLE) mainly depended on the activation of NFAT1 signaling.^[^ ^25^ ^]^ NFAT1 is a key transcription factor for regulating T cell differentiation, activation, exhaustion, and self‐tolerance.^[^ ^27^, ^31^, ^44^ ^]^ Cytoplasmic NFAT1 is phosphorylated in resting T cells and dephosphorylated after activation, which translocates to the nucleus and targets the promoter/enhancer regions of T cell activation genes to induce an increasing number of T cell activation markers and effector cytokines.^[^ ^31^ ^]^ Other studies showed that the IL‐15/STAT5 signaling pathway also closely associates with CD154 expression.^[^ ^45^, ^46^ ^]^ High serum IL‐15 than that in healthy controls is explored in the SLE patients, which activated STAT5 and subsequently participated in the prolonged CD154 expression.^[^ ^23^ ^]^ Here, we demonstrated that NFAT1 in activating CD4+ T cells exhibited depolarization and translocation to the nucleus, and STAT5 was also activated and phosphorylated after PMA and ionomycin stimulation. However, UC‐MSC‐EVs could limit NFAT1 activation, but not STAT5, and attenuate CD154 expression. Moreover, NFAT1 is a calcium‐dependent protein. Since CD4+ T cell activation, the activity of calcium channels on the cell membrane was upregulated and the rapidly elevated amount of cytoplasmic Ca^2+^ binds with calmodulin (CaM) to trigger the activity of calcineurin coupling with nuclear transportation of NFAT1 protein.^[^ ^28^, ^47^ ^]^ Calcineurin is required for the depolarization and maintenance of NFAT1 activity in the nucleus depended on intracellular Ca^2+^ stimulation. Interestingly, our findings in this study suggest that MSC‐EVs modulate the function of Ca^2+^ channels so that they affect the concentration of Ca^2+^ and the expression of calcineurin of CD4+ T cells.

Increasing evidence indicates that EVs are a main bioactive component of MSCs to participate in anti‐inflammation with their contents of relevant proteins, mRNAs and microRNAs. To further explore the molecular mechanism of UC‐MSC‐EVs modulating Ca^2+^ influx of CD4+ T cells, we detected proteomics for the definition of UC‐MSC‐EVs. Among proteins identification, we focused on CCT2 for further analysis as its reported roles on regulating the activity of Ca^2+^ channels.^[^ ^34^ ^]^ Chaperonin containing TCP1 (CCT) is a large ring complex composed of eight homologous subunits, which is identified to facilitate the folding of 5–10% newly synthesized proteins and was initially discovered as an assistant for folding tubulin and actin to functional 3D configuration.^[^ ^48^, ^49^, ^50^, ^51^, ^52^ ^]^ Chaperonin is traditionally divided into two groups, including Group I that was found in chloroplasts, mitochondria, and bacteria and Group II in the cytoplasm in eukaryotic cells.^[^ ^53^ ^]^ CCT is also a conserve protein that is mainly encoded on the T complex gene cluster on chromosome 17.^[^ ^54^ ^]^ Recent studies revealed the novel effect of CCT on modulating the activity of Ca^2+^ channels.^[^ ^35^ ^]^ CCT2 is a subunit of CCT. Hodeify et al. utilized knockdown CCT2 cell line to demonstrate that CCT2 significantly suppresses the activity of Ca^2+^ channel Orai1 by regulating Orai1 endocytosis, but not affecting their expression.^[^ ^34^ ^]^ In detail, CCT2 could interact with Orai1 through its intercellular loop and decrease Orai1 residence in plasma membrane, resulting in weakened Ca^2+^ signaling as well as NFAT1 inactivation.^[^ ^34^ ^]^ To further confirm the proteomics results in this study, we found that the plasma membrane residence of Orai1 was decreased while the total Orai1 was little affected in CD4+ T cells after uptake of MSC‐EVs, which was consistent with the previous findings. In addition, we knockdowned CCT2 expression of UC‐MSCs and harvested UC‐MSC‐EVs^shCCT2^. Knockdown of CCT2 significantly weakened the effect of UC‐MSC‐EVs on restricting Ca^2+^ influx in CD4+ T cells, accompanied with the higher NFAT1 translocating to nucleus and the higher CD154 expression on CD4+ T cells than that of UC‐MSC‐EVs. Moreover, treatment of liver IRI model with UC‐MSC‐EVs^shCCT2^ increased the reduced expression of membranous CD154 of the intrahepatic CD4+ T cells so that reversed the hepatoprotective effect of EVs, indicating that UC‐MSC‐EVs exert their immunoregulative role to affect CD154 synthesis and expression mainly via transferring CCT2 to CD4+ T cells in liver IRI model. Besides CCT2, UC‐MSC‐EVs also contain many other proteins through our proteomics analysis. Therefore, further studies are warranted to detect whether other proteins are involved in this immunoregulative role.

**Figure 8:**
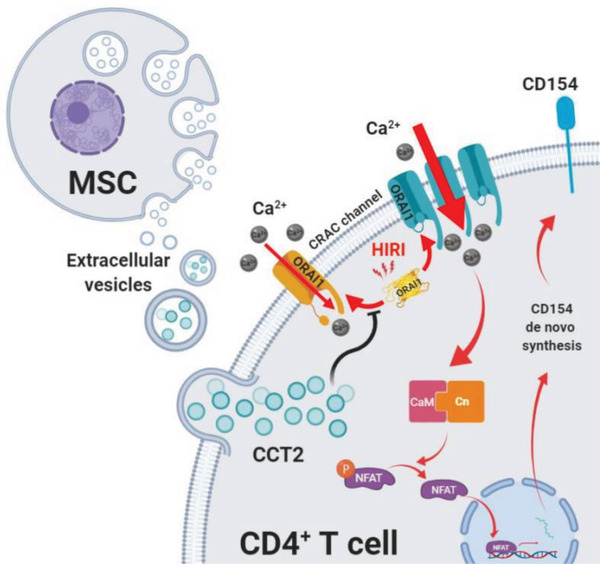
Hepatoprotective and immunoregulative effects of UC‐MSC‐EVs. Schematic diagram showing the potential molecular mechanisms through which UC‐MSC‐EVs alleviate hepatocellular damage by downregulating membranous CD154 expression of intrahepatic CD4+ T cells and limiting the initiation of inflammatory response after IRI.

In conclusion, this study took advantage of in vivo and in vitro experiments to show the novel mechanisms through which UC‐MSCs attenuate liver IRI. The mechanisms underlying the effects of these cells under IRI condition might involve CCT2 in EVs, which were secreted by UC‐MSCs, to downregulate CD154 expression on the intrahepatic CD4+ T cells through restricted Ca^2+^‐calcineurin‐NFAT1 pathway (**Figure** **8**). These results further suggest that EVs are a critical component for MSCs to play bioactive roles in regulating CD4+ T cells functions, which is an attractive therapeutic approach for preventing the initiation of the inflammatory response in liver IRI.

## 4 Experimental Section

##### UC‐MSC‐EVs Isolation

The procedures of isolating UC‐MSCs were approved by the Research Ethics Committee of the Third Affiliated Hospital of Sun Yat‐sen University. EVs were harvested from the UC‐MSC supernatant as previously described.^[^ ^14^ ^]^ In brief, UC‐MSCs (the fourth generation) were seeded and cultured in T225 cm^2^ flasks with low‐glucose Dulbecco's modified Eagle medium (DMEM) and 10% fetal bovine serum (FBS). Until reached 70–80% confluence, the medium was replaced with low‐glucose DMEM containing 10% exosome‐depleted FBS and the cells were cultured for another 48 h. Subsequently, the supernatant was collected and centrifuged at 3200 *g* for 10 min at 4 °C to pellet the residual cells. Then, the samples were transferred to ultracentrifuge tubes and centrifuged at 10 000 *g* for 30 min at 4 °C to remove the cellular debris. The UC‐MSC‐EVs were harvested by ultracentrifugation twice at 100 000 *g* for 2 h at 4 °C. After removing supernatant, the EVs were saved in 100 µL PBS and stored at −80 °C. To detect the total protein content of collected EVs, a bicinchoninic protein assay kit (KeyGEN BioTECH, Jiangsu, China) was used according to the manufacturer's protocol.

##### Nanoparticle Tracking Analysis

Size distribution of UC‐MSC‐EVs was determined by NTA using a NanoSight LM10 instrument (Malvern Instruments Ltd, Malvern, UK), equipped with a sCMOS camera. Data were analyzed with the NTA software version 3.1.54, with detection threshold set to 5, and blur and Max Jump Distance set to auto. Samples were diluted ten times with PBS to reach optimal concentration for instrument linearity. Readings were taken on once of per 10 s, at a camera level set to 16 and with manual monitoring of temperature.

##### Cellular Uptake of UC‐MSC‐EVs

To detect the uptake of UC‐MSC‐EVs by CD4+ T cells, UC‐MSCs‐EVs were first labeled with 10^−6^ m PKH26 Cell Membrane Labeling Dye (Sigma‐Aldrich, USA) following the manufacturer's protocol. After quenched by PBS containing 10% bovine serum albumin (BSA), the PKH26 labeled EVs were harvested by ultracentrifugation at 100 000 *g* for 2 h at 4 °C. Labeled UC‐MSC‐EVs were added to the CD4+ T cells and co‐cultured for 2 h. Then, the cells were washed twice with PBS and fixed with 4% paraformaldehyde (PFA, pH 7.4) for 30 min at 4 °C. The fixative cells were washed with PBS and placed on the glass slide. After performing the immunofluorescent staining procedures with CD4+ T cells marker (CD4 primary antibody, 1:200, Abcam, USA), the fluorescent intensity was investigated under a confocal microscope (LSM880, Zeiss, Germany).

##### Animals

C57BL/6, B6 mice (male, 8–10 weeks old, weighting 22–25 g) were purchased from the Guangdong Medical Laboratory Animal Center (Guangdong, China). All mice were housed in the Experimental Animal Center of Sun Yat‐sen University under specific pathogen‐free (SPF) conditions and were given care according to the Guideline of Sun Yat‐sen University for Animal Experimentation. Animals were received free access to standard laboratory diet and water, which were maintained at constant environment, 50% humidity and 20 °C temperature, 12 h dark and light cycle.

##### Model of Liver IRI and In Vivo Experimental Design

All experimental processes of animal were abided by the National Institutes of Health Guide for the Care and Use of Laboratory Animals and were allowed from the Animals Care and Used Committee of the Third Affiliated Hospital of Sun Yat‐sen University (Guangzhou, China). The standard protocol of a mouse liver IRI model was conducted as previously described.^[^ ^55^, ^56^ ^]^ In brief, after analgesia by intraperitoneally injecting 1% pentobarbital sodium (100 µL/10 g), an atraumatic vascular clip was used to occlude the portal vein and hepatic artery from 70% liver, including the left lateral and median lobes of liver. Ischemic period was sustained for 90 min. Then, the atraumatic vascular clamp was removed to finish the ischemic stage. In addition, a midline laparotomy incision was performed without any other procedures, which was considered as the sham group.

After establishment of the liver IRI model, mice were randomly divided into two groups, which were presented or absented in UC‐MSC‐EVs (100 µg/100 µL). In addition, two groups were further expanded, namely, the liver IRI mice were treated with UC‐MSC‐EVs^shCCT2^ or UC‐MSC‐EVs^NC‐siRNA^, respectively. All of the experimental mice were sacrificed at 6 h after reperfusion.

##### Tracking of UC‐MSC‐EVs In Vivo

To monitor the biodistribution of UC‐MSC‐EVs in vivo, the fluorescent lipophilic tracer DiR‐labeled EVs were used immediately to peripheral intravenous inject after reperfusion, with control mice treating with DiR supernatant which was collected after DiR‐labeled UC‐MSC‐EVs isolation. The processes of labeling UC‐MSC‐EVs were performed as previously described.^[^ ^57^ ^]^ In brief, the pelleting UC‐MSC‐EVs were incubated with DiR (1 × 10^−6^ m, Invitrogen, Life Technologies, USA) for 15 min at room temperature in the dark. After quenching PBS with 10% BSA, the DiR‐labeled EVs were isolated by ultracentrifugation as mentioned above. And the supernatant was collected as the control. The images of UC‐MSC‐EVs distribution in vivo were, respectively, taken 3 and 6 h later using the Bruker Small Animal Optical Imaging System (In‐Vivo Xtreme II; Billerica, MA). The organs, including heart, lung, liver, spleen, and kidney, were also harvested at the related time‐points and labeled‐EVs were quantitated ex situ using the same imaging system.

Furthermore, pathological examination was performed to monitor the localization of labeled‐EVs. The organs from exo‐red UC‐MSC‐EVs‐injected ischemic mice were collected and fixed in 4% PFA for 1 h. After that, the tissues were dehydrated by sucrose gradient and embedded in optimal cutting temperature (OCT) compound until the tissues were loaded at the bottom of the gradient. 20 µm liver sections were made using freezing microtome (Thermo Fisher Scientific, USA) and stained with DAPI (4′,6‐diamidino‐2‐phenylindole) for 2 min in the dark. The slides were mounted using Prolong Diamond Antifade Mounting Agent (Life, USA) and observed under a fluorescent microscope (Leica, Germany).

##### Fluorescent TUNEL

Fluorescent TUNEL was conducted with a commercial in situ apoptosis detection kit (Roche Diagnostics, Indianapolis, IN, USA) on 20 µm thick cryosections according to the manufacturer's instruction. In brief, the enzyme and label solution were mixed into the TUNEL reaction mixture. Then, the cryosections were treated with equilibration buffer and exposed to the reaction mixture for 60 min at 37 °C in the dark. The slides were incubated in DAPI for 2 min and mounted by Prolong Diamond Antifade Mounting Agent. The sections were observed and photographed under a fluorescent inverted microscope (Leica, Germany) at 20× magnification. The results were processed and analyzed using Image J (NIH, USA).

##### Immunofluorescent Staining

To analyze the number of macrophages in the liver tissues of each group, immunofluorescent staining was performed as previously described.^[^ ^58^ ^]^ Briefly, 4 µm thick liver sections were dewaxed, rehydrated, repaired by ethylene diamine tetraacetic acid (EDTA, pH 8.0), and incubated with primary antibody (CD68, 1:200, Abcam, USA) at 4 °C overnight. Alexa Flour 594‐conjugated secondary antibody (1:1000, Life, USA) was treated subsequently for 1 h at room temperature in the dark. Then, DAPI was used to nucleus staining at room temperature for 2 min. The sections were observed and photographed under a fluorescent inverted microscope (Leica, Germany).

##### Isolation of Intrahepatic Mononuclear Cells

Intrahepatic mononuclear cell isolation was performed as previously described.^[^ ^59^ ^]^ In brief, the liver was perfused in situ through portal vein with Hank's balanced salt solution (HBSS, without calcium and magnesium, Gibco, Australia) to remove intrahepatic blood. Then, the liver was minced into 1 mm^3^ pieces in HBSS (with calcium and magnesium) containing collagenase H (Roche, Switzerland) and digested for 20 min at 37 °C. RPMI‐1640 containing with 10% heat‐inactivated FBS was used to terminate digestion, and the tissues were then passed through 70 µm cell strainers. After centrifugation at 380 *g* for 10 min at 4 °C, the deposits were resuspended in 40% Percoll (GE Healthcare, USA) and mildly overlaid onto 70% Percoll. The intrahepatic mononuclear cells were collected from the interface layer after centrifugation at 870 *g* for 30 min.

##### Flow Cytometry Analysis

Flow cytometry analysis was applied to detect the expression of CD154 on CD4+ T cells in in vivo and in vitro study. For surface antibodies staining, the collected mononuclear cells isolated from livers and the cells from in vitro experiments were incubated with fluorescence‐conjugate anti‐mouse antibodies against CD3 (FITC, BioLegend), CD4 (PE, BD), and CD154 (APC, BD) for 30 min at 4 °C. The fluorescence of the cells was detected using an eight‐color FACS Calibur (BD, USA).

##### CD4+ T Cells Isolation and In Vitro Experimental Design

The CD4+ T cells used in in vitro experiments were prepared from the spleen of C57BL/6 mice by positive selection using a mouse CD4+ T cells MicroBeads UltraPure kit (Miltenyi Biotec, Germany) according to the manufacturer's protocol. Flow cytometry was used to determine the purity of CD4+ T cells, which was more than 95%.

*Group design*: To explore the mechanism that how UC‐MSC‐EVs regulate CD154 expression on CD4+ T cells, in vitro model was also established, which was randomly divided into six groups, including: normal group, CD4+ T cells were cultured in the typical medium without any intervention; Control group, CD4+ T cells were stimulated by PMA (MultiSciences, Hangzhou, China) and ionomycin (MultiSciences) without any other treatment; UC‐MSC‐CM group, CD4+ T cells were incubated with PMA and ionomycin and treated with UC‐MSC‐CM; UC‐MSC‐ECM group, UC‐MSC‐CM was replaced by UC‐MSC‐ECM; UC‐MSC‐EV group, CD4+ T cells were subjected to PMA and ionomycin stimulation as well as treated with UC‐MSC‐EVs; GSK‐5498A group, CD4+ T cells were treated with GSK‐5498A (calcium channel blocker, 1 × 10^−6^ m, MedChem Express, Monmouth Junction, NJ, USA)^[^ ^33^ ^]^; UC‐MSC‐EV^shCCT2^ group, CD4+ T cells were treated with UC‐MSC‐EVs^shCCT2^ and incubated in the medium containing PMA and ionomycin.

##### Preparation of Nuclear, Cytosolic, and Membranous Fraction

Nuclear and cytosolic proteins were extracted using NE‐PER Nuclear and Cytoplasmic Extraction Reagents (Thermo Scientific, USA) according to the manufacturer's instruction. In brief, the cells were harvested and centrifugated at 500 *g* for 5 min. After washed with PBS, the cell pellet was added with ice‐cold cytoplasmic extraction reagent (CER) I, vortexed for 15 s to fully suspend the cell pellet, and incubated on ice for 10 min. After adding ice‐cold CER II, the cells were, then, vortexed for 5 s, incubated on ice for 1 min, vortexed for another 5 s, and centrifuged at 16 000 *g* for 5 s. The supernatant was collected as cytosolic fraction. Subsequently, the insoluble fraction was suspended in ice‐cold nuclear extraction reagent and vortexed for 15 s. After that, the sample was placed on ice and continually vortexed for 15 s every 10 min, a total of 40 min. Finally, centrifugation was performed at 16 000 *g* for 10 min, and this supernatant was also harvested as nuclear fraction. To isolate membrane protein fractions of cultured cells, Mem‐PER Plus Membrane Protein Extraction Kit (Thermo Scientific) was utilized following the manufacturer's protocol.

##### Confocal Microscopy

After stimulation, mouse primary T cells were fixed with 4% PFA at room temperature for 30 min, air‐dried on microscopic glass slides for 2 h, blocked with 5% goat serum for 30 min, and incubated with primary antibody (anti‐NFAT1, 1:200) overnight. After washed three times with PBS for 5 min, the cells were treated with species‐matched secondary antibody conjugated with fluorochrome dye Alexa 594 (1:1000) at room temperature for 1 h in the dark. The cell nuclei were stained with DAPI for 2 min and subsequently observed and photographed under a Zeiss 880 confocal microscope (Nikon Instruments, Melville, New York, United States). 10–15 fields were randomly chosen per slide and analyzed using ImageJ.

##### Intracellular Ca^2+^ Detection

The Ca^2+^ concentration of CD4+ T cells was measured to detect few minor changes from each group. After treatment according to the group design mentioned above, the cells were incubated in HBSS containing 4 × 10^−6^ m Fluo4‐AM (Solarbio Science & Technology, Beijing, China) at 37 °C for 20 min and, then, added with HBSS with 1% fetal calf serum to incubate for another 40 min. Subsequently, the cells were washed and bathed in HEPES (4‐(2‐hydroxyethyl)‐1‐piperazineethanesulfonic acid) buffer saline (10 × 10^−3^ m HEPES, 1 × 10^−3^ m Na_2_HPO_4_, 137 × 10^−3^ m NaCl, 5 × 10^−3^ m KCl, 1 × 10^−3^ m CaCl_2_, 0.5 × 10^−3^ m MgCl_2_, 5 × 10^−3^ m Glucose, and 0.1% BSA) with pH modulated to 7.4 for 10 min. Fluorescence images of the cells from each group were detected and photographed under a Zeiss 880 confocal microscope. The maximum emission wavelength of Fluo4 fluorescence was 516 nm, and the maximum excitation wavelength was 494 nm. The ratio of fluorescence was obtained using multifunctional enzyme mark (Tecan Spark 10M, Austria).

##### Co‐Immunoprecipitation

The samples were lysed in 4 °C cold lysis buffer (containing HEPES (10 × 10^−3^ m, pH 8.0), EDTA (0.1 × 10^−3^ m), glycerol (20%), NP‐40 (0.2%), NaCl (300 × 10^−3^ m), and protease inhibitor) for 30 min. The lysates were collected after centrifugation at 10 000 *g* for 5 min, and cleared via incubating with protein A/G agarose (Gibco, Carlsbad, CA) at 4 °C for 30 min. The supernatant was incubated with an indicated antibody and placed in horizontal shaker at 4 °C overnight. Subsequently, the lysates were treated with the protein G beads at 4 °C for 60 min. Finally, the samples were washed with cold lysis buffer, added with the protein loading buffer, and boiled at 95 °C for 5 min, followed by Western blot assay.

##### Plasmid Construction and Knockdown CCT2 by Lentivirus Infection

Short‐hairpin RNAs (shRNAs) lentiviral transduction particles targeting the sequence of CCT2 was used to interfere the expression of the CCT2 gene of UC‐MSCs. In brief, CCT2 shRNA (SHCLNV‐NM‐006431) and control shRNA were purchased from Sigma‐Aldrich (USA).^[^ ^60^ ^]^ According to the manufacturer's protocol, UC‐MSCs were infected with CCT2 shRNA or control shRNA at a multiplicity of infection of 5. After 48 h incubation, infected cells were chosen by transferring the cells in selection media containing puromycin (Sigma, USA) for another 72 h. Lentiviral interference efficiency was confirmed using Western blotting assay. To assess cell viability, UC‐MSCs and UC‐MSCs^shCCT2^ were, respectively, cultured 24, 48, 72, 96, and 120 h, the cells were collected and detected in a hemocytometer (Neubauer Improved Bright Line Hemacytometer, Marien Feld, Germany) after stained with 0.4% trypan blue.

## 5 Statistical Analysis

The data are presented as the mean ± standard error of mean (SD) or as values directly. Statistical analysis of results was conducted by one‐way analysis of variance (ANOVA) or unpaired two‐tailed Student's *t*‐test, as appropriate. All statistical analyses were performed using GraphPad Prism 7 software (GraphPad Software, San Diego, CA). A probability level of *p*‐value <0.05 or <0.01 was considered statistically significant.

## Conflict of Interest

The authors declare no conflict of Interest.

## Supporting information

Supporting Information

## References

[1] Y. Zhai , H. Petrowsky , J. C. Hong , R. W. Busuttil , J. W. Kupiec‐Weglinski , Nat. Rev. Gastroenterol. Hepatol. 2013, 10, 79. 23229329 10.1038/nrgastro.2012.225 PMC3577927

[2] A. Casillas‐Ramirez , I. B. Mosbah , F. Ramalho , J. Rosello‐Catafau , C. Peralta , Life Sci. 2006, 79, 1881. 16828807 10.1016/j.lfs.2006.06.024

[3] O. Motino , D. E. Frances , N. Casanova , M. Fuertes‐Agudo , C. Cucarella , J. M. Flores , M. T. Vallejo‐Cremades , L. Olmedilla , J. Perez Pena , R. Banares , L. Bosca , M. Casado , P. Martin‐Sanz , Hepatology 2019, 70, 650. 30155948 10.1002/hep.30241

[4] J. Kahn , P. Schemmer , Visc. Med. 2018, 34, 444. 30675491 10.1159/000493889 PMC6341346

[5] K. Oishi , S. Hagiwara , S. Koga , S. Kawabe , T. Uno , H. Iwasaka , T. Noguchi , J. Surg. Res. 2012, 176, 164. 22560539 10.1016/j.jss.2011.03.080

[6] X. Chen , C. Cai , D. Xu , Q. Liu , S. Zheng , L. Liu , G. Li , X. Zhang , X. Li , Y. Ma , L. Huang , J. Chen , J. Shi , X. Du , W. Xia , A. P. Xiang , Y. Peng , Theranostics 2019, 9, 16. 10.7150/thno.32260 PMC6643430 31367246

[7] J. Panes , D. Garcia‐Olmo , G. Van Assche , J. F. Colombel , W. Reinisch , D. C. Baumgart , A. Dignass , M. Nachury , M. Ferrante , L. Kazemi‐Shirazi , J. C. Grimaud , F. de la Portilla , E. Goldin , M. P. Richard , A. Leselbaum , S. Danese , A. C. S. G. Collaborators , Lancet 2016, 388, 1281. 27477896 10.1016/S0140-6736(16)31203-X

[8] J. Zheng , H. Li , L. He , Y. Huang , J. Cai , L. Chen , C. Zhou , H. Fu , T. Lu , Y. Zhang , J. Yao , Y. Yang , Cell Proliferation 2019, 52, e12546. 30537044 10.1111/cpr.12546 PMC6496237

[9] H. S. Kim , D. Y. Choi , S. J. Yun , S. M. Choi , J. W. Kang , J. W. Jung , D. Hwang , K. P. Kim , D. W. Kim , J. Proteome Res. 2012, 11, 2. 10.1021/pr200682z 22148876

[10] F. Collino , M. C. Deregibus , S. Bruno , L. Sterpone , G. Aghemo , L. Viltono , C. Tetta , G. Camussi , PLoS One 2010, 5, e11803. 20668554 10.1371/journal.pone.0011803 PMC2910725

[11] Z. Du , C. Wei , J. Yan , B. Han , M. Zhang , C. Peng , Y. Liu , Liver Transplant. 2013, 19, 215. 10.1002/lt.23577 23193024

[12] S. R. Baglio , D. M. Pegtel , N. Baldini , Front. Physiol. 2012, 3, 359. 22973239 10.3389/fphys.2012.00359 PMC3434369

[13] Y. Shi , Y. Wang , Q. Li , K. Liu , J. Hou , C. Shao , Y. Wang , Nat. Rev. Nephrol. 2018, 14, 493. 29895977 10.1038/s41581-018-0023-5

[14] J. Yao , J. Zheng , J. Cai , K. Zeng , C. Zhou , J. Zhang , S. Li , H. Li , L. Chen , L. He , H. Chen , H. Fu , Q. Zhang , G. Chen , Y. Yang , Y. Zhang , FASEB J. 2019, 33, 2. 10.1096/fj.201800131RR 30226809

[15] X. Shen , Y. Wang , F. Gao , F. Ren , R. W. Busuttil , J. W. Kupiec‐Weglinski , Y. Zhai , Hepatology 2009, 50, 1537. 19670423 10.1002/hep.23153 PMC2805281

[16] X. Shen , F. Reng , F. Gao , Y. Uchida , R. W. Busuttil , J. W. Kupiec‐Weglinski , Y. Zhai , Am. J. Transplant. 2010, 10, 1729. 20659085 10.1111/j.1600-6143.2010.03205.x PMC3655759

[17] L. Ding , J. M. Green , C. B. Thompson , E. M. Shevach , J. Immunol. 1995, 155, 11. 7594521

[18] B. O. Lee , L. Haynes , S. M. Eaton , S. L. Swain , T. D. Randall , J. Exp. Med. 2002, 196, 693. 12208883 10.1084/jem.20020845 PMC2194000

[19] G. S. Hassan , J. Stagg , W. Mourad , Cancer Treat. Rev. 2015, 41, 431. 25843228 10.1016/j.ctrv.2015.03.007

[20] T. Seijkens , D. Engel , M. Tjwa , E. Lutgens , Thromb. Haemostasis 2010, 104, 639. 10.1160/TH10-03-0174 20694278

[21] U. Schonbeck , P. Libby , Cell. Mol. Life Sci. 2001, 58, 4. 11229815 10.1007/PL00000776 PMC11146501

[22] G. Z. Pan , Y. Yang , J. Zhang , W. Liu , G. Y. Wang , Y. C. Zhang , Q. Yang , F. X. Zhai , Y. Tai , J. R. Liu , Q. Zhang , G. H. Chen , J. Surg. Res. 2012, 178, 935. 22658855 10.1016/j.jss.2012.04.070

[23] R. M. Lowe , A. Genin , N. Orgun , R. Q. Cron , Genes Immun. 2014, 15, 137. 24500400 10.1038/gene.2014.3 PMC4133980

[24] S. A. Crist , D. L. Sprague , T. L. Ratliff , Blood 2008, 111, 3553. 18180380 10.1182/blood-2007-05-088161 PMC2275021

[25] S. F. Wu , C. B. Chang , J. M. Hsu , M. C. Lu , N. S. Lai , C. Li , C. H. Tung , Arthritis Res. Ther. 2017, 19, 183. 28793932 10.1186/s13075-017-1393-y PMC5550984

[26] Y. Zhang , R. B. Liu , Q. Cao , K. Q. Fan , L. J. Huang , J. S. Yu , Z. J. Gao , T. Huang , J. Y. Zhong , X. T. Mao , F. Wang , P. Xiao , Y. Zhao , X. H. Feng , Y. Y. Li , J. Jin , J. Clin. Invest. 2019, 129, 7. 10.1172/JCI123801 PMC6597231 31135381

[27] G. N. Huang , D. L. Huso , S. Bouyain , J. Tu , K. A. McCorkell , M. J. May , Y. Zhu , M. Lutz , S. Collins , M. Dehoff , S. Kang , K. Whartenby , J. Powell , D. Leahy , P. F. Worley , Science 2008, 319, 5862. 10.1126/science.1151227 PMC3602998 18218901

[28] F. Macian , Nat. Rev. Immunol. 2005, 5, 472. 15928679 10.1038/nri1632

[29] C. K. Go , R. Hooper , M. R. Aronson , B. Schultz , T. Cangoz , N. Nemani , Y. Zhang , M. Madesh , J. Soboloff , Sci. Signaling 2019, 12, eaaw2627. 10.1126/scisignal.aaw2627 PMC6800072 31594854

[30] V. C. Kyttaris , Z. Zhang , O. Kampagianni , G. C. Tsokos , Arthritis Rheum. 2011, 63, 2058. 21437870 10.1002/art.30353 PMC3128171

[31] P. G. Hogan , Cell Calcium 2017, 63, 66. 28153342 10.1016/j.ceca.2017.01.014 PMC5739523

[32] D. E. Clapham , Cell 2007, 131, 1047. 18083096 10.1016/j.cell.2007.11.028

[33] J. Chen , M. J. Sanderson , J. Physiol. 2017, 595, 10. 10.1113/JP272694 PMC5430234 27396568

[34] R. Hodeify , M. Nandakumar , M. Own , R. J. Courjaret , J. Graumann , S. Z. Hubrack , K. Machaca , Sci. Adv. 2018, 4, eaau1935. 30263962 10.1126/sciadv.aau1935 PMC6157965

[35] H. Yang , M. Hu , J. Guo , X. Ou , T. Cai , Z. Liu , Nature 2016, 538, 537. 27698420 10.1038/nature19767

[36] Z. Du , C. Wei , K. Cheng , B. Han , J. Yan , M. Zhang , C. Peng , Y. Liu , J. Surg. Res. 2013, 183, 907. 23522455 10.1016/j.jss.2013.02.009

[37] J. Rao , F. Cheng , S. Yang , Y. Zhai , L. Lu , Cell Mol. Immunol. 2019, 16, 98. 29907880 10.1038/s41423-018-0051-x PMC6318264

[38] J. H. Lai , S. F. Luo , L. J. Ho , Cells 2019, 8, 8.

[39] J. Mehta , A. Genin , M. Brunner , L. V. Scalzi , N. Mishra , T. Beukelman , R. Q. Cron , Arthritis Rheum. 2010, 62, 2499. 20506525 10.1002/art.27554 PMC2921031

[40] Y. C. Zhang , W. Liu , B. S. Fu , G. Y. Wang , H. B. Li , H. M. Yi , N. Jiang , G. Wang , J. Zhang , S. H. Yi , H. Li , Q. Zhang , Y. Yang , G. H. Chen , Cytotherapy 2017, 19, 2. 10.1016/j.jcyt.2016.11.005 27964826

[41] Y. Fan , F. Herr , A. Vernochet , B. Mennesson , E. Oberlin , A. Durrbach , Stem Cells Dev. 2019, 28, 44. 30328799 10.1089/scd.2018.0015

[42] Y. Liu , G. Lou , A. Li , T. Zhang , J. Qi , D. Ye , M. Zheng , Z. Chen , EBioMedicine 2018, 36, 140. 30197023 10.1016/j.ebiom.2018.08.054 PMC6197728

[43] M. Monguio‐Tortajada , S. Roura , C. Galvez‐Monton , J. M. Pujal , G. Aran , L. Sanjurjo , M. Franquesa , M. R. Sarrias , A. Bayes‐Genis , F. E. Borras , Theranostics 2017, 7, 270. 28042333 10.7150/thno.16154 PMC5197063

[44] G. J. Martinez , R. M. Pereira , T. Aijo , E. Y. Kim , F. Marangoni , M. E. Pipkin , S. Togher , V. Heissmeyer , Y. C. Zhang , S. Crotty , E. D. Lamperti , K. M. Ansel , T. R. Mempel , H. Lahdesmaki , P. G. Hogan , A. Rao , Immunity 2015, 42, 265. 25680272 10.1016/j.immuni.2015.01.006 PMC4346317

[45] L. Baranda , H. de la Fuente , E. Layseca‐Espinosa , D. Portales‐Perez , P. Nino‐Moreno , G. Valencia‐Pacheco , C. Abud‐Mendoza , J. Alcocer‐Varela , R. Gonzalez‐Amaro , Rheumatology 2005, 44, 1507. 16251219 10.1093/rheumatology/kei083

[46] M. Aringer , G. H. Stummvoll , G. Steiner , M. Koller , C. W. Steiner , E. Hofler , H. Hiesberger , J. S. Smolen , W. B. Graninger , Rheumatology 2001, 40, 876. 11511756 10.1093/rheumatology/40.8.876

[47] G. R. Crabtree , E. N. Olson , Cell 2002, 109, S67. 11983154 10.1016/s0092-8674(02)00699-2

[48] J. Frydman , E. Nimmesgern , H. Erdjument‐Bromage , J. S. Wall , P. Tempst , F. U. Hartl , EMBO J. 1992, 11, 4767. 1361170 10.1002/j.1460-2075.1992.tb05582.x PMC556952

[49] Y. Gao , J. O. Thomas , R. L. Chow , G. H. Lee , N. J. Cowan , Cell 1992, 69, 1043. 1351421 10.1016/0092-8674(92)90622-j

[50] C. Spiess , A. S. Meyer , S. Reissmann , J. Frydman , Trends Cell Biol. 2004, 14, 598. 15519848 10.1016/j.tcb.2004.09.015 PMC2812437

[51] C. Dekker , P. C. Stirling , E. A. McCormack , H. Filmore , A. Paul , R. L. Brost , M. Costanzo , C. Boone , M. R. Leroux , K. R. Willison , EMBO J. 2008, 27, 1827. 18511909 10.1038/emboj.2008.108 PMC2486426

[52] A. Y. Yam , Y. Xia , H. T. Lin , A. Burlingame , M. Gerstein , J. Frydman , Nat. Struct. Mol. Biol. 2008, 15, 1255. 19011634 10.1038/nsmb.1515 PMC2658641

[53] A. L. Horwich , W. A. Fenton , E. Chapman , G. W. Farr , Annu. Rev. Cell Dev. Biol. 2007, 23, 115. 17489689 10.1146/annurev.cellbio.23.090506.123555

[54] M. Sugimoto , Genes Genet. Syst. 2014, 89, 3. 25475932 10.1266/ggs.89.97

[55] A. Castellaneta , O. Yoshida , S. Kimura , S. Yokota , D. A. Geller , N. Murase , A. W. Thomson , Hepatology 2014, 60, 267. 24493010 10.1002/hep.27037 PMC4077928

[56] H. L. Van Sweringen , N. Sakai , R. C. Quillin , J. Bailey , R. Schuster , J. Blanchard , H. Goetzman , C. C. Caldwell , M. J. Edwards , A. B. Lentsch , Hepatology 2013, 57, 331. 22961770 10.1002/hep.26049 PMC3540195

[57] H. Haga , I. K. Yan , D. A. Borrelli , A. Matsuda , M. Parasramka , N. Shukla , D. D. Lee , T. Patel , Liver Transplant. 2017, 23, 791. 10.1002/lt.24770 PMC5495137 28407355

[58] R. M. Egeler , B. E. Favara , J. D. Laman , E. Claassen , Eur. J. Cancer 2000, 36, 2105. 11044648 10.1016/s0959-8049(00)00296-3

[59] M. Swamydas , Y. Luo , M. E. Dorf , M. S. Lionakis , Curr. Protoc. Immunol. 2015, 110, 3.20.1. 26237011 10.1002/0471142735.im0320s110 PMC4574512

[60] M. Kasembeli , W. C. Lau , S. H. Roh , T. K. Eckols , J. Frydman , W. Chiu , D. J. Tweardy , PLoS Biol. 2014, 12, e1001844. 24756126 10.1371/journal.pbio.1001844 PMC3995649

