# Supplementary material for: Extracellular Vesicles Derived from Human Umbilical Cord Mesenchymal Stem Cells Protect Liver Ischemia/Reperfusion Injury by Reducing CD154 Expression on CD4+ T Cells via CCT2
Source: Adv Sci (Weinh). 2020 Aug 20;7(18):1903746. doi: 10.1002/advs.201903746 (PMC7509664; doi:10.1002/advs.201903746)
Supplement: Supplementary file 1 — Supporting Information [file ADVS-7-1903746-s001.pdf]

© 2020 Wiley-VCH GmbH

ADVANCED SCIENCE
Open Access

Supporting Information

for *Adv. Sci.,* DOI: 10.1002/advs.201903746

Extracellular Vesicles Derived from Human Umbilical Cord Mesenchymal Stem Cells Protect Liver Ischemia/Reperfusion Injury by Reducing CD154 Expression on CD4+ T Cells via CCT2

Jun Zheng, Tongyu Lu, Chaorong Zhou, Jianye Cai, Xiaomei Zhang, Jinliang Liang, Xin Sui, Xiaoyan Chen, Liang Chen, Yao Sun, Jiebin Zhang, Wenjie Chen, Yingcai Zhang, Jia Yao,* Guihua Chen,* and Yang Yang**

Supporting Information

Extracellular Vesicles Derived from Human Umbilical Cord Mesenchymal Stem Cell Protect Liver Ischemia/Reperfusion Injury by Reducing CD154 Expression on CD4+ T Cells via CCT2

Jun Zheng, Tongyu Lu, Chaorong Zhou, Jianye Cai, Xiaomei Zhang, Jinliang Liang, Xin Sui, Xiaoyan Chen, Liang Chen, Yao Sun, Jiebin Zhang, Yingcai Zhang*, Jia Yao*, Guihua Chen*, Yang Yang*

Isolation, culture and identification of umbilical cord-derived MSCs (UC-MSCs)

The procedures of isolating UC-MSCs have been proved by the Research Ethics Committee of the Third Affiliated Hospital of Sun Yat-sen University. The standard procedures were performed following to the previous study.[1] UC-MSCs were isolated and cultured under aseptic maintenance. Umbilical cords (UCs) were collected after donor agreed with signing the consent inform and washed twice using phosphate-buffer saline (PBS) to wipe out the remnant blood. The UCs were cut into

10 mm^3/piece and placed in type I collagenase containing with hyaluronidase (0.1%) and $CaCl_2$ (3mM). After 4 h digestion at 37℃, the UCs were transferred into low-glucose(1 g/L) Dulbecco's Modified Eagle Medium (DMEM, Gibco, Life, Australia) basic medium with 10% fetal bovine serum (FBS, Gibco, Australia) and cultured in the humidified atmosphere with 5% CO_2 at 37℃. Medium was refreshed every 3 days to remove nonadherent cells.

To identify the characterizations of UC-MSCs and UC-MSCs^{shCCT2}, the potentials of adipogenesis and osteogenesis were also detected to assess their multipotential differentiation according to previously described.[2]

Harvest of UC-MSC conditioned medium and EV-depleted conditioned medium

Complete conditioned medium (CM) from UC-MSC (UC-MSC-CM) was prepared as previous described.[3] In brief, since grown to 70-80%, the cells were washed twice with PBS and cultured in low- glucose DMEM with 10% exosome-depleted FBS for another 48 h. The UC-MSC-CM was harvested and centrifugated at 3,200 g 4℃ for 10 min to remove debris. To collect EV-depleted condition medium from UC-MSC (UC-MSC-ECM), the UC-MSC-CM was ultra-centrifugated at 100,000 for 2 h to deplete human EVs.[1] Finally, the supernatant was obtained and filtered through a filter (0.22 μm).

Transmission Electron Microscope

The size and morphology of UC-MSC-EVs were detected by transmission electron microscope (TEM). The specimens were prepared by placing UC-MSC-EVs on a copper mesh and stained with 3% phosphotungstic acid (Ph 7.0) at 2 min. The specimens were observed and photographed under a H-7650 TEM (HITACHI High-Technologies Corp., Tokyo, Japan) at an accelerating voltage of 80 kV.

Detection of liver function

Serum alanine aminotransferase (ALT), aspartate aminotransferase (AST) and lactate dehydrogenase (LDH), which are indicators of liver injury, were measured by a 7180 Biochemical Analyzer (Hitachi, Japan).

Histological evaluation of liver section

4 μm thick liver paraffin sections were prepared and stained with hematoxylin-eosin (H&E) to determine liver injury. The severity of IRI in liver tissues was scored blindly according to the Suzuki's criteria which was classified 0-4 scale.[4] The scale was evaluated by three pathological indicators, including hepatocyte necrosis, ballooning degeneration and sinusoidal congestion. No centrilobular ballooning, hepatocellular necrosis and congestion is given 0, while the area of necrosis >60% and severe ballooning degeneration and congestion is given 4.

Enzyme-Linked Immunosorbent Assay (ELISA)

The concentrations of IFN-γ and TNF-α in serum and liver tissue in vivo study and in supernatant in vitro study were measured by enzyme-linked immunosorbent assay kits following to the manufacturer's protocols (EIAAB Science Company, Wuhan, China). The optical density (OD) was determined at 450 nm by an automatic microplate reader (Biotek Vermont, USA).

Western blotting

After equated the amounts of proteins, 20 μg sample was subjected to 10% SDS polyacrylamide gel electrophoresis (PAGE) and, then, transferred onto polyvinylidene difluoride membranes (PVDF) (Millipore, Billerica, MA, USA). The membranes were immersed in 5% non-fat milk on oscillating table at room

temperature for 1 h to block non-specific antigen and respectively treated with the primary antibodies at 4 ℃ overnight. Subsequently, the membranes were incubated with the secondary antibody (anti-rabbit IgG, 1:5000, Sigma-Aldrich) at room temperature for 1 h. After treated with an enhanced chemiluminescence (ECL) substrate, the blots were visualized by a FluorChem System imager (ProteinSimple, CA, USA). The intensities of the blots were analyzed using an image analyzer (ImageJ software, USA).

Total RNA extraction and quantitative real-time polymerase chain reaction (RT-qPCR)

Total RNA extraction and qRT-PCR were performed according to the previous described.[5] In brief, total RNA extractions from the both UC-MSCs and liver tissue were used TRIzol (Invitrogen) following to the manufacturer's instruction. After checking the amount and purity of total RNA according to the absorbance at 260 nm and 280 nm wavelengths using Biophotometer plus (Eppendorf, Germany), cDNA was reverse transcribed using Transcriptor First Strand cDNA Synthesis Kit (Roche Applied Science, USA). cDNA amplification was performed with heating at 65℃ for 10 min firstly, incubating at 55℃ for 30min sequentially, then deactivating at 85℃ for 5min and storage at 4℃ 5 min, at last, using the PCR Thermal Cycler (Bio-Rad, USA). Finally, RT-PCR was performed with Roche Applied Science SYBR Master Mix using reverse transcription system (LC-480, Roche, USA). β-actin, as a housekeeping gene, were used to normalization. Specific primer sequences used to amplify in this study are listed in Supplemental Table 1.

Supplemental Table 1. Primer sequences for quantitative RT-PCR

Gene Symbol	Sequence direction	Sequence

Mouse-IFN-γ	Forward primer	5'- GCGTCATTGAATCACACCTG -3'
	Reverse primer	5'- TGAGCTCATTGAATGCTTGG -3'
Mouse-IL-6	Forward primer	5'- TCCATCCAGTTGCCTTCTTG -3'
	Reverse primer	5'- CCACGATTTCCCAGAGAACA -3'
Mouse-TNF-α	Forward primer	5'- AGCACAGAAAGCATGATCCG -3'
	Reverse primer	5'- CTGATGAGAGGGAGGCCATT -3'
Mouse-β-actin	Forward primer	5'- GTTGTCGACGACGAGCG -3'
	Reverse primer	5'- GCACAGAGCCTCGCCTT -3'

Proteomic analysis by Nano-LC-MS/MS

Label-free LC-MS/MS experiment: For label-free experiment, UC-MSC-EVs were lysed and their proteins were extracted by total protein extraction kit (KeyGENE, Jiangsu, China). Equal volume of proteins was separated on 12.5% SDS-PAGE gel, of which the Coomassie Blue staining (Bio-Red, USA) was used to visualize the protein bands. For protein digestion, filter-aided sample preparation (FASP) method was performed as previous described.[6] In simple terms, protein sample was suspended in 200 μl UA buffer (8 M urea, 150 mM Tris-HCl, pH 8.0; Sigma) to incubate in room temperature for 1 h. Then, 100 μl of 10 mM iodoacetamide (Sigma) was added and incubated in the dark for 30 min. Next, the sample was washed twice with 200 μl UA buffer at 14,000 g centrifugation for 10 min at room temperature. And the sample was added with 50 μl trypsin working solution (5 μg of trypsin dissolving in 50 μl of ultrapure water) for digesting and incubating at 37℃ overnight. The digested samples were desalted on C18 Cartridges (Empore™ SPE Cartridges C18, bed I.D. 7 mm, volume 3 ml; Sigma), concentrated by centrifugation at 14,000 g for 10 min and reconstituted in 40 μl of 0.1% formic acid. For LC-MS/MS analysis, an Easy-Nlc1000 Liquid Chromatograph coupled to a Q Exactive mass spectrometer (Thermo Fisher Scientific, USA) was conducted for 120 min. Peptides were separated on a RP-C18 analytical column at a flow rate of 300 nl/min over 120 min. After the capillary separation, digested samples were analyzed using a Q Exactive mass spectrometer.

Data calculation and analysis: The raw data was carried out in Mascot software (http://www.matrixscience.com/; version 2.6) and searched against Uniprot_HomoSapiens_20386_20180905. The search parameters were set as following: enzyme: trypsin, maximum missed cleavages:2, instrument: ESI-TRAP, precursor mass tolerance: ±10 ppm, fragment mass tolerance: 0.05 Da, use average precursor mass: false, modification groups from Quan method: TMT 6 plex, dynamic modifications: oxidation; acetyl, static modifications, database pattern: decoy, peptide FDR: ≤0.01. The expressed proteins obtained from each sample in each group were interested by groups, and the interesting expressed proteins were used for bioinformatics analysis. Gene Ontology (GO) and KEGG were annotated and enriched with the R-package 'clusterprofier'[7], and Reactome was annotated and enriched with the R-package 'ReactomePA'[34]. For functional annotation and enrichment analysis of Biocarta, Biocarta database was extracted from the R-package 'pathfinder'[35] and analyzed using 'clusterProfier' package`.

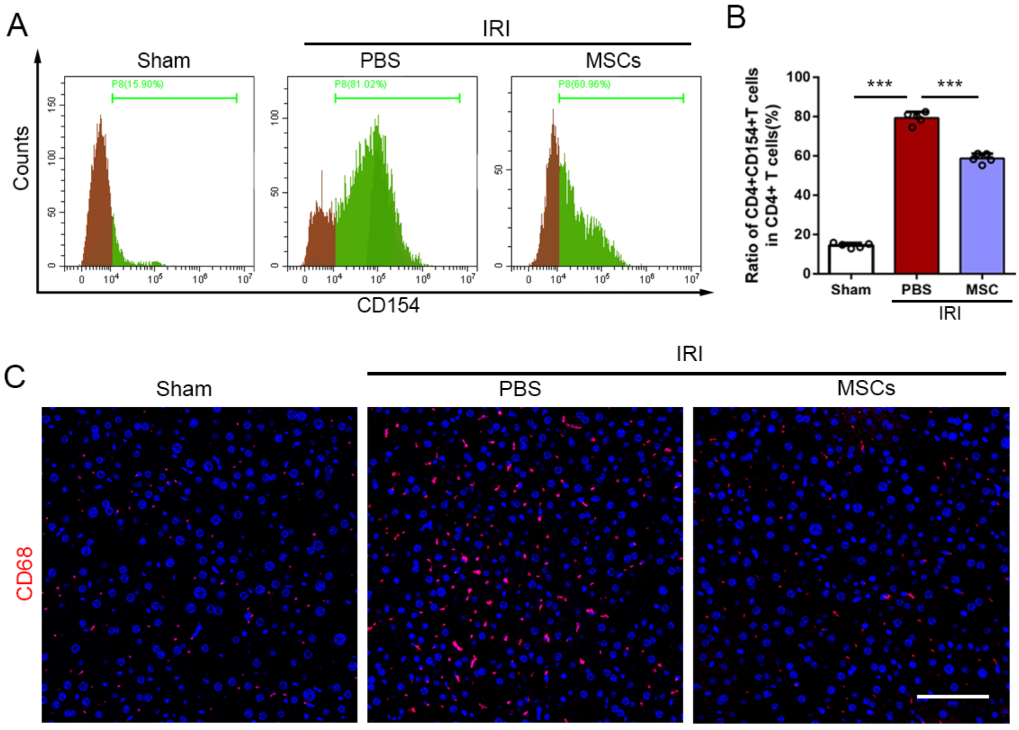

Supplemental Figure 1. The effect of UC-MSCs on regulating inflammatory responses in liver tissues of the liver IRI mice.

Treatment with UC-MSCs or PBS to the liver IRI mice were sacrificed after 6 h of reperfusion. (A) The peak diagram of flow cytometry analyses of membranous CD154 expression of intrahepatic CD4+ T cells in each group. (B) Quantification of membranous CD154 expression of intrahepatic CD4+ T cells. The data are presented as the means ± SEM (n=5/group). (C) Representative liver sections from each group stained with fluorescent CD68 (red fluorescence) (Bar = 200 μm). *$p<0.05$, **$p<0.01$, ***$p<0.001$ (all p values were obtained by one-way ANOVA).

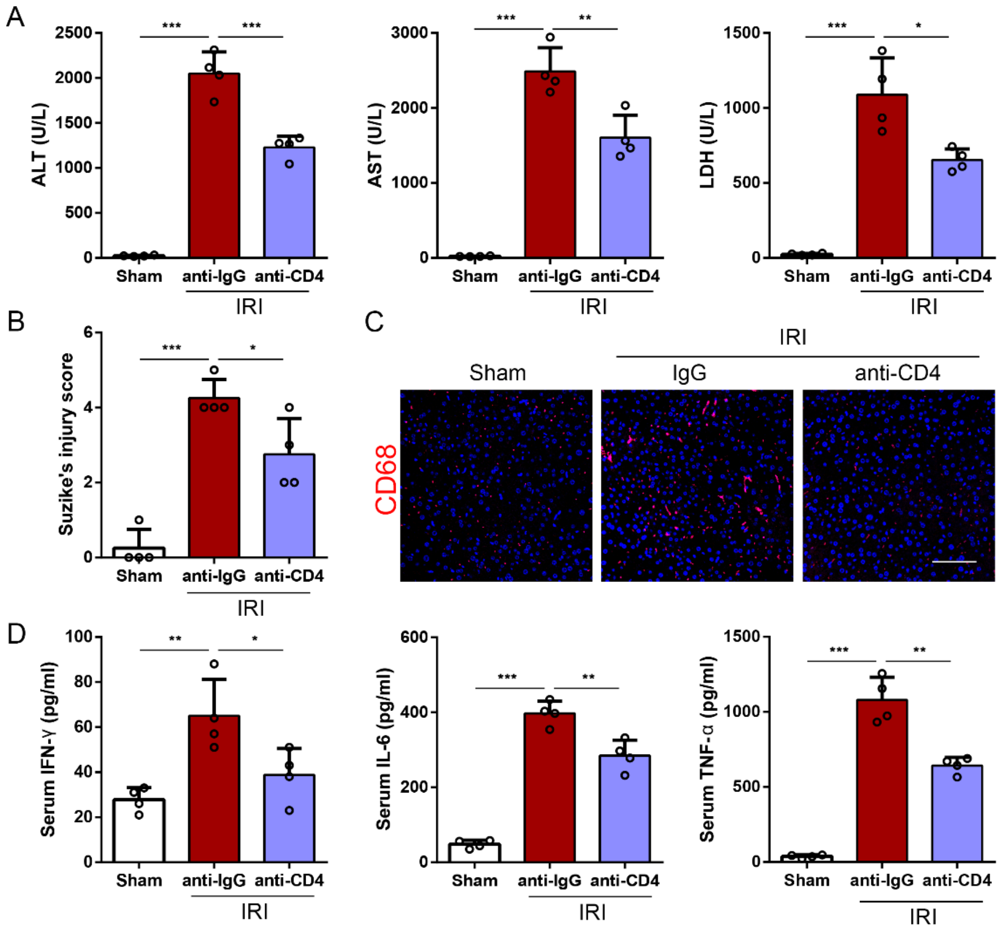

Supplemental Figure 2. CD4+ T cells are pivotal for the initiation of inflammatory response in liver IRI

Mice underwent liver IRI were pre-treated with CD4 depleting Ab (GK1.5) and anti-IgG antibody (as control), and then were sacrificed at 6 h after reperfusion. (A) Serum ALT, AST and LDH from normal control (Sham), anti-IgG-treated liver IRI mice and anti-CD4-treated liver IRI mice were detected, respectively. Data are presented as the means ± SEM (n =5 mice/groups). (B) Statistical analyses of the Suzike's injury score of each group were performed, which were used to determine the degree of liver injury. Data are presented as the means ± SEM (n=5 mice/group). (C) Representative fluorescence images of CD68 staining (red fluorescence) from each group (Bar = 200 μm). (D) The levels of IFN-γ, IL-6 and TNF-α in serum from each group were measured using ELISA assay. Data

are presented as the means ± SEM (n=5 mice/group). *p<0.05, **p<0.01, ***p<0.001 (all p values were obtained by one-way ANOVA).

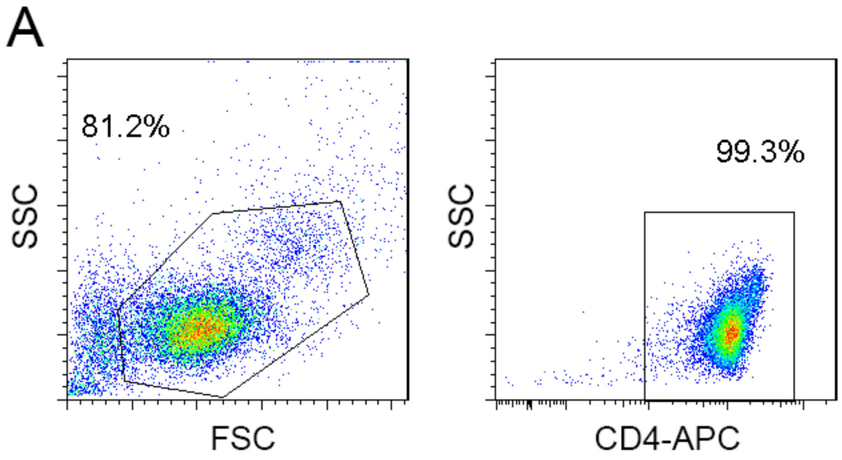

Supplemental Figure 3. The purity of isolated CD4+ T cells from the spleen of mice.

(A) The purity of CD4+ T cells that were isolated from the spleen of mice using a MicroBeads UltraPure kit was determined by flow cytometry analysis.

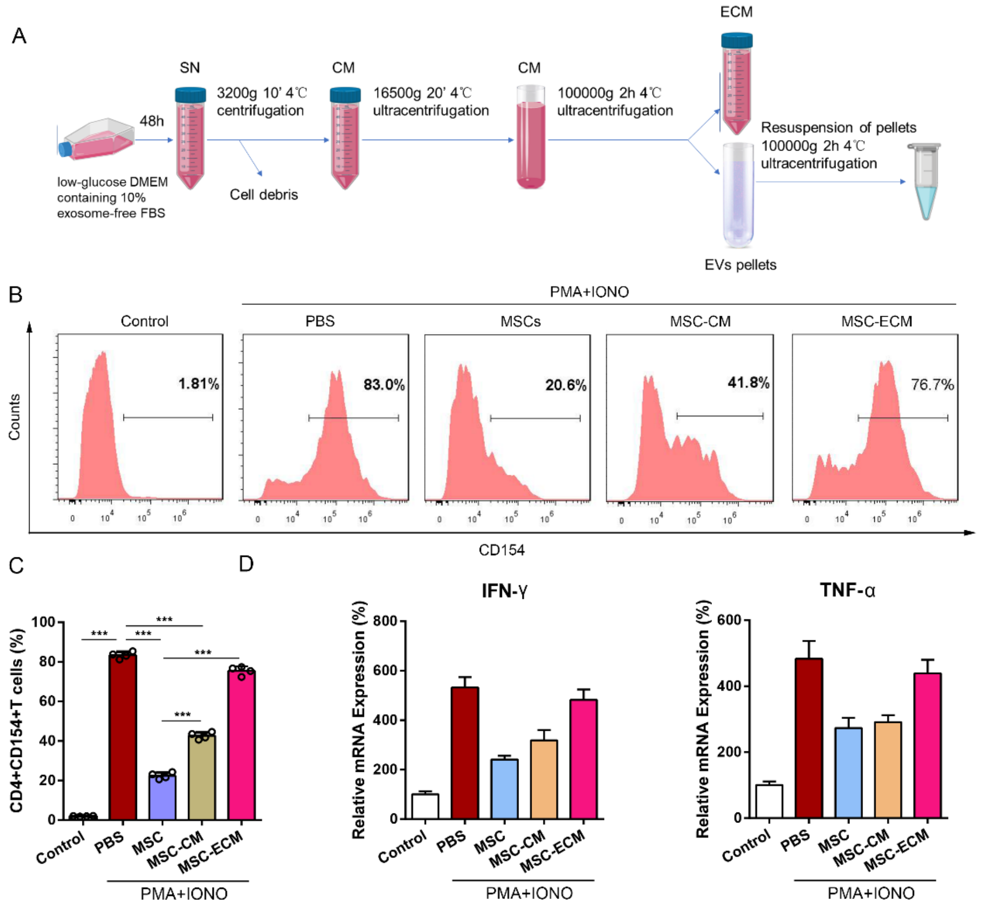

Supplemental Figure 4. UC-MSC-CM suppresses CD4+ T cells inflammatory cytokines production and CD154 expression in vitro

Primary CD4+ T cells isolated from the spleen of mice were stimulated by PMA and ionomycin, and co-cultured with PBS, UC-MSCs, UC-MSC-CM or UC-MSC-ECM. (A) The methodological procedure for obtaining UC-MSC-CM, UC-MSC-ECM and UC-MSC-EVs from UC-MSC 48 h-culture. (B) Flow cytometry analyses of CD154 expression on CD4+ T cells of each treatment group. (C) Quantification of membranous CD154 expression of CD4+ T cells. The data are presented as the means ± SEM (n=3 /group). (D) The levels of mRNA expression of IFN-γ and TNF-α in CD4+ T cells from each group were determined by RT-qPCR. Data are presented as mean±SEM (n=3/group). *p<0.05, **p<0.01, ***p<0.001 (all p values were obtained by one-way ANOVA).

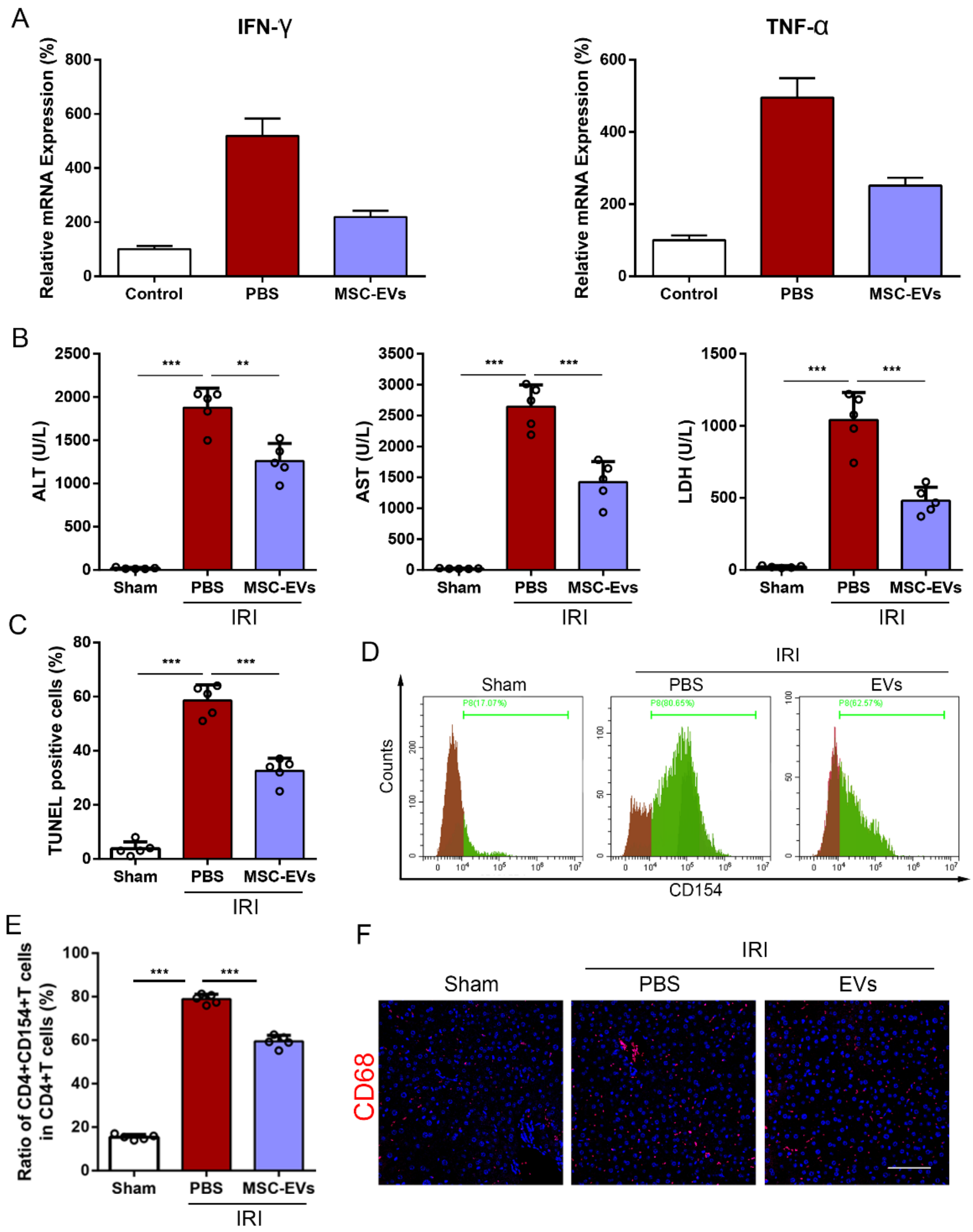

Supplemental Figure 5. UC-MSC-EVs modulate CD4+ T cells function and attenuate liver IRI

In vitro experiments, primary CD4+ T cells isolated from the spleen of mice were stimulated by PMA and ionomycin, and co-cultured with PBS or UC-MSC-EVs. (A) The levels of mRNA expression of IFN-γ and TNF-α in CD4+ T cells from each group were determined by RT-qPCR. Data are presented as mean±SEM (n=3/group). Mice that underwent liver IRI were treated with PBS or UC-MSC-EVs and sacrificed at 6 h after reperfusion. (B) Serum ALT, AST and

LDH from the normal control (Sham group), PBS-treated liver IRI mice, and UC-MSC-EVs-treated liver IRI mice were detected. The data are expressed as the means±SEMs (n=5/group). (C) Statistical analyses of the percent of TUNEL positive cells in each section. Data are presented as the means ± SEM (n=5 mice/group). (D) The peak diagram of flow cytometry analyses of membranous CD154 expression of intrahepatic CD4+ T cells in each group. (E) Quantification of membranous CD154 expression of intrahepatic CD4+ T cells. The data are presented as the means ± SEM (n=5/group). (F) Representative liver sections from each group stained with fluorescent CD68 (red fluorescence) (Bar = 200 μm). *p<0.05, **p<0.01, ***p<0.001 (all p values were obtained by one-way ANOVA).

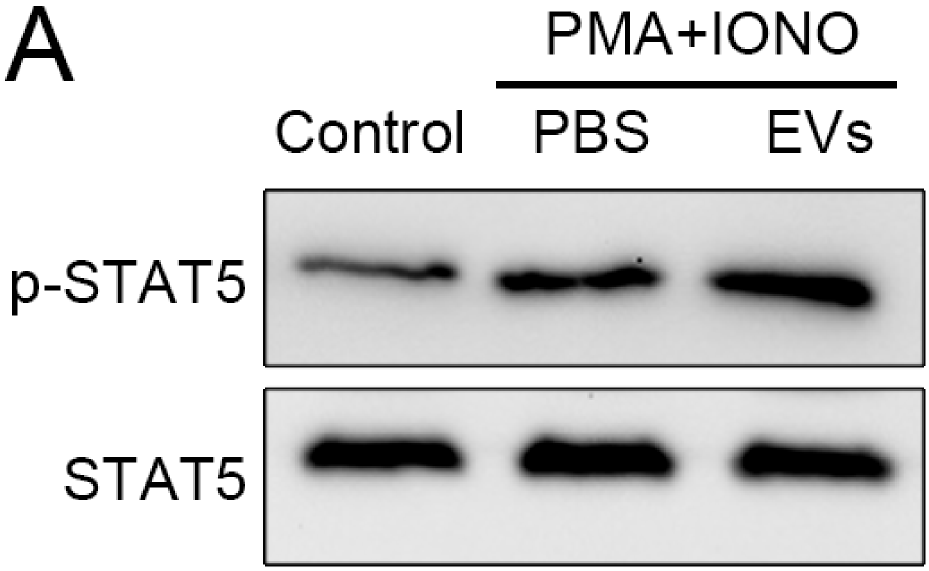

Supplemental Figure 6. The effect of UC-MSC-EVs on modulating membranous CD154 expression of CD4+ T cells did not target STAT5.

Primary CD4+ T cells isolated from the spleen of mice were stimulated by PMA and ionomycin, and co-cultured with PBS or UC-MSC-EVs. (A) The activation of STAT5 (p-STAT5) in CD4+ T cells was detected by Western blotting assay.

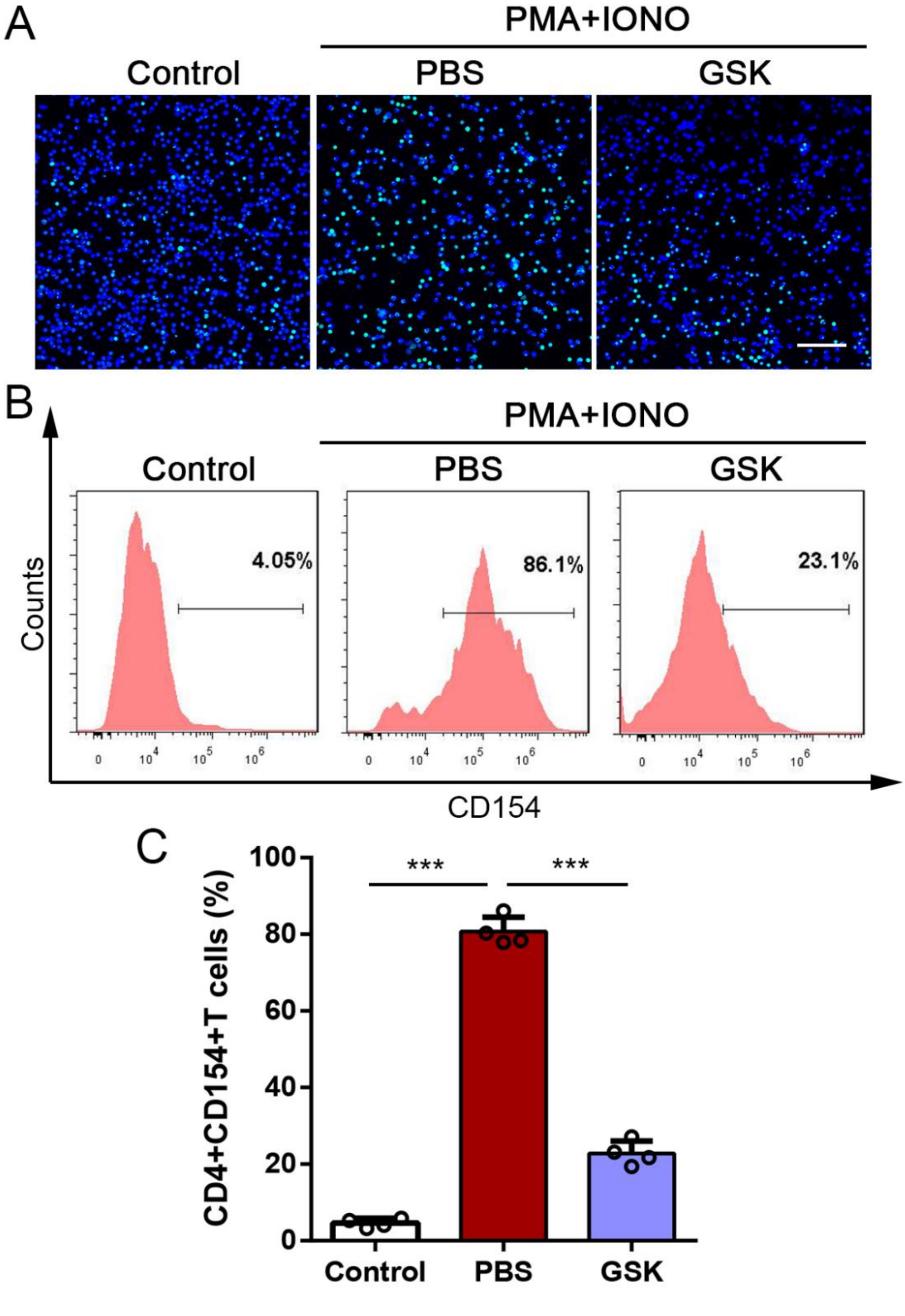

Supplemental Figure 7. The effect of GSK-5498A on modulating membranous CD154 expression of CD4+ T cells.

Primary CD4+ T cells isolated from the spleen of mice were stimulated by PMA and ionomycin, and co-cultured with PBS or GSK-5498A. (A) Representative

images of Ca^{2+} influx dynamics in PBS- or UC-MSC-EVs-treated CD4+ T cells using Fluo4 fluorescence (Bar = 60 μm). (B) Flow cytometry analyses of CD154 expression on CD4+ T cells of each treatment group. (C) Quantification of membranous CD154 expression of CD4+ T cells. The data are presented as the means ± SEM (n=3 /group). *p<0.05, **p<0.01, ***p<0.001 (all p values were obtained by one-way ANOVA).

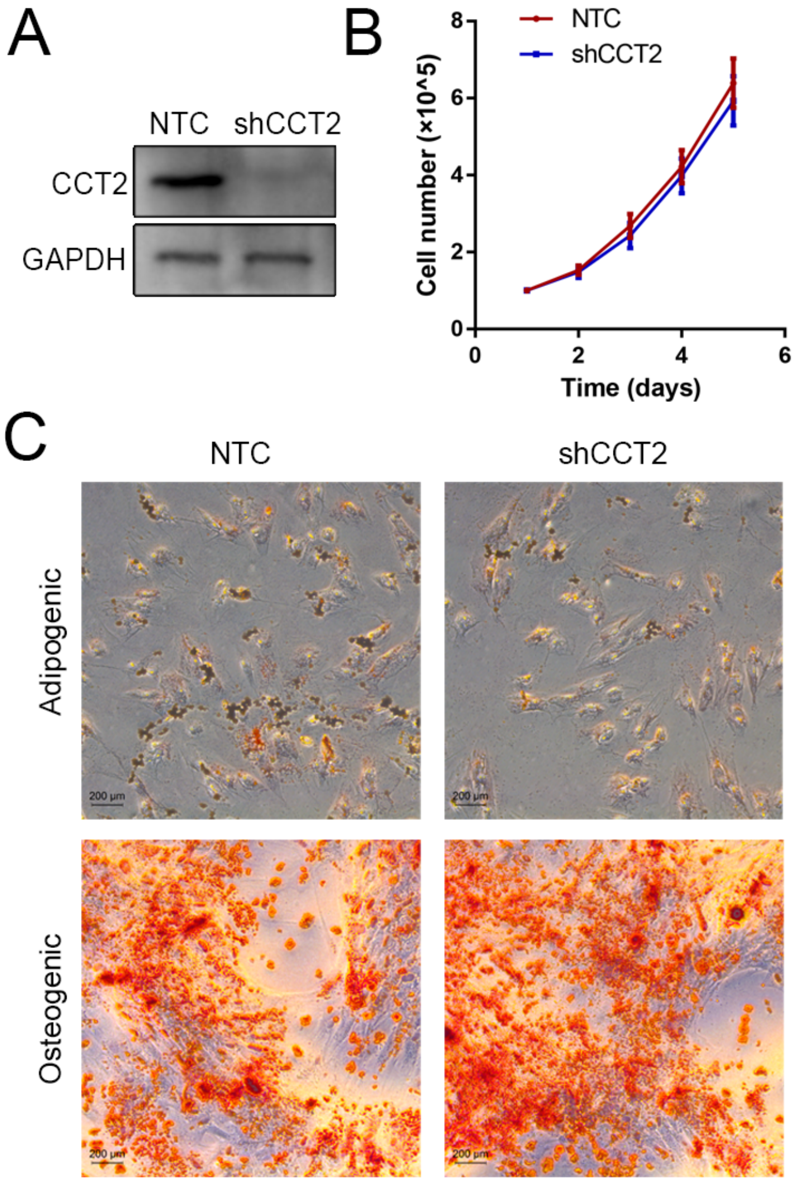

Supplemental Figure 8. The lentivirus carrying *CCT2*-shRNA was

successfully transfected to UC-MSCs

(A) To ascertain the effect of the siRNA on CCT2 expression in UC-MSCs, CCT2 expression was evaluated by Western blot analysis. (B) UC-MSCs and UC-MSCs^{shCCT2} were counted after incubated for 1, 2, 3, 4 and 5 days to evaluate cell viability. The data are presented as the means ± SEM (n=3 /group). (C) Representative pictures of UC-MSCs and UC-MSCs^{shCCT2}, which differentiate into adipogenesis and osteogenesis after 21 d specific culture. *p<0.05, **p<0.01, ***p<0.001 (all p values were obtained by one-way ANOVA).

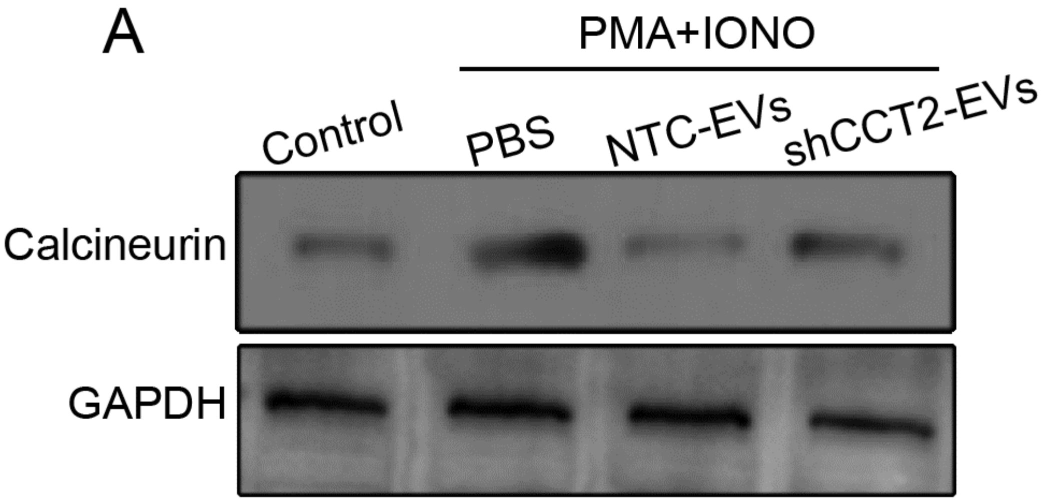

Supplemental Figure 9. CCT2 derived from UC-MSC-EVs modulated calcineurin expression of CD4+ T cells

Primary CD4+ T cells isolated from the spleen of mice were stimulated by PMA and ionomycin, and co-cultured with PBS, MSC-EVs, or UC-MSCs^{shCCT2}. (A) The expression of calcineurin in CD4+ T cells in each group was determined by Western blot analysis. The average intensity of the band in the Western blots was quantified using GAPDH as an internal reference.

Reference

[1] M. Monguio-Tortajada, S. Roura, C. Galvez-Monton, J. M. Pujal, G. Aran, L. Sanjurjo, M. Franquesa, M. R. Sarrias, A. Bayes-Genis, F. E. Borras, *Theranostics* **2017**, *7*, 2.

[2] J. Yao, J. Zheng, J. Cai, K. Zeng, C. Zhou, J. Zhang, S. Li, H. Li, L. Chen, L. He, H. Chen, H. Fu, Q. Zhang, G. Chen, Y. Yang, Y. Zhang, *FASEB J* **2019**, *33*, 2.

[3] G. Z. Pan, Y. Yang, J. Zhang, W. Liu, G. Y. Wang, Y. C. Zhang, Q. Yang, F. X. Zhai, Y. Tai, J. R. Liu, Q. Zhang, G. H. Chen, *J Surg Res* **2012**, *178*, 2.

[4] C. Fondevila, X. D. Shen, S. Tsuchiyashi, K. Yamashita, E. Csizmadia, C. Lassman, R. W. Busuttil, J. W. Kupiec-Weglinski, F. H. Bach, *Hepatology* **2004**, *40*, 6.

[5] S. Saraswat, P. K. Rout, S. D. Kharche, S. K. Jindal, A. K. Goel, *Reprod Domest Anim* **2016**, *51*, 6.

[6] J. R. Wisniewski, N. Nagaraj, A. Zougman, F. Gnad, M. Mann, *J Proteome Res* **2010**, *9*, 6.

[7] G. Yu, L. G. Wang, Y. Han, Q. Y. He, *OMICS* **2012**, *16*, 5.

[8] G. Yu, Q. Y. He, *Mol Biosyst* **2016**, *12*, 2.

[9] E. Ulgen, O. Ozisik, O. U. Sezerman, *Front Genet* **2019**, *10*,
